# An ancestral molecular response to nanomaterial particulates

**DOI:** 10.1038/s41565-023-01393-4

**Published:** 2023-05-08

**Authors:** G. del Giudice, A. Serra, L. A. Saarimäki, K. Kotsis, I. Rouse, S. A. Colibaba, K. Jagiello, A. Mikolajczyk, M. Fratello, A. G. Papadiamantis, N. Sanabria, M. E. Annala, J. Morikka, P. A. S. Kinaret, E. Voyiatzis, G. Melagraki, A. Afantitis, K. Tämm, T. Puzyn, M. Gulumian, V. Lobaskin, I. Lynch, A. Federico, D. Greco

**Affiliations:** 1 FHAIVE, Faculty of Medicine and Health Technology, Tampere University, Tampere, Finland; 2 Tampere Institute for Advanced Study, Tampere, Finland; 3 School of Physics, University College Dublin, Dublin, Ireland; 4 Group of Environmental Chemoinformatics, Faculty of Chemistry, University of Gdańsk, Gdańsk, Poland; 5 QSAR Lab Ltd, Gdańsk, Poland; 6 School of Geography, Earth and Environmental Sciences, University of Birmingham, Birmingham, UK; 7 Novamechanics Ltd, Nicosia, Cyprus; 8 National Institute for Occupational Health, National Health Laboratory Services, Johannesburg, South Africa; 9 School of Health Systems and Public Health, University of Pretoria, Pretoria, South Africa; 10 Institute of Biotechnology, Helsinki Institute of Life Sciences (HiLife), University of Helsinki, Helsinki, Finland; 11 Division of Physical Sciences and Applications, Hellenic Military Academy, Vari, Greece; 12 Institute of Chemistry, University of Tartu, Tartu, Estonia; 13 Haematology and Molecular Medicine Department, School of Pathology, University of the Witwatersrand, Johannesburg, South Africa; 14 Water Research Group, Unit for Environmental Sciences and Management, North West University, Potchefstroom, South Africa; 15 Division of Pharmaceutical Biosciences, Faculty of Pharmacy, University of Helsinki, Helsinki, Finland

**Keywords:** Nanoparticles, Nanobiotechnology

## Abstract

The varied transcriptomic response to nanoparticles has hampered the understanding of the mechanism of action. Here, by performing a meta-analysis of a large collection of transcriptomics data from various engineered nanoparticle exposure studies, we identify common patterns of gene regulation that impact the transcriptomic response. Analysis identifies deregulation of immune functions as a prominent response across different exposure studies. Looking at the promoter regions of these genes, a set of binding sites for zinc finger transcription factors C_2_H_2_, involved in cell stress responses, protein misfolding and chromatin remodelling and immunomodulation, is identified. The model can be used to explain the outcomes of mechanism of action and is observed across a range of species indicating this is a conserved part of the innate immune system.

## Abstract (web summary)

While engineered nanomaterials are relatively new, organisms have been exposed to natural nanoparticles over vast periods of time. Here the authors explore the possibility that common mechanisms of response to nanomaterials may have resulted from a long evolutionary exposure history to natural nano-sized matter.

## Main

Nano-toxicogenomics aims at unravelling the potential toxicity of engineered nanomaterials (ENMs). To date, a plethora of transcriptomics data has been generated for this purpose^1^.

Finding commonalities among environmental exposures allows grouping of ENM by mechanism of action (MOA), which would streamline their safety assessment^2^.

However, because of the high complexity and variability of systemic responses to ENMs, transcriptomic profiles are heterogeneous and typically biased by small-scale datasets (low numbers of ENM, limited doses and time points). This results in a myriad of toxicogenomic signatures, with low similarity to each other (Supplementary Fig. 1).

The heterogeneity of the ENM transcriptomic signatures hampers the possibility to highlight commonalities between in vitro responses and real-life exposure scenarios.

Improving in vitro–in vivo extrapolation requires the definition of models able to transpose the mechanisms of toxicity from shorter observations in vitro (hours or days) to long timescales (weeks or months) in vivo^3^.

In contrast to the specificity of the transcriptional response to environmental signals, the regulation of gene expression is usually well conserved across species^4^.

The environment modulates the epigenome^5^, and several studies suggested that epigenetic signals can stably maintain modulation well beyond the duration of the stimulus (adaptive plasticity)^6^. Transcriptional changes usually follow an ‘impulse-like’ kinetic, while regulation of transcription is achieved by multiple layers of sustained epigenetic signals^7^. For this, gene regulation is associated with more stable physiopathological changes and can detect exposure-induced alteration more reliably than transcript changes alone.

**Fig. 1:**
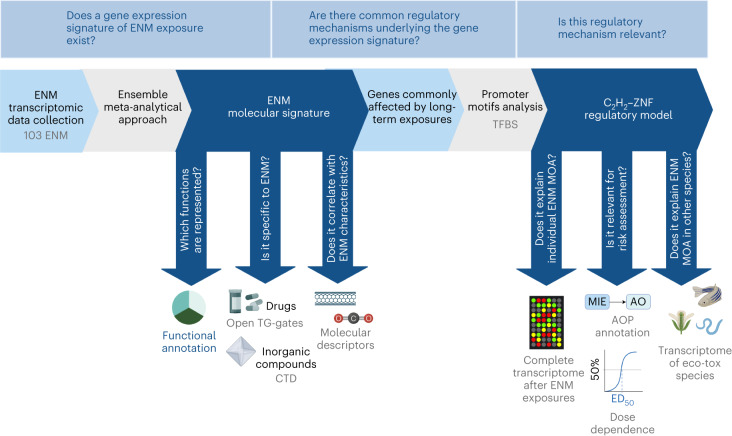
Study workflow. Study workflow, including the data used, their sources (grey text) and methodological steps (light grey arrow boxes and blue text). The light blue boxes at the top of the figure indicate the hypothesis underpinning the study. AO, adverse outcome. ED_50_, mediant effective dose.

In this Analysis, we hypothesized that common patterns of gene regulation underlie the response of multiple biological systems exposed to a variety of ENMs (Fig. 1). We analysed the most comprehensive transcriptomics data collection for ENMs to date, in which the expression of 3,676 genes is measured across 584 experimental conditions^8–10^. This collection includes multiple human and mouse cell types and tissues, both in vitro and in vivo, exposed to 103 ENMs varying in chemistry, geometry and size (Supplementary Fig. 2a).

## An ENM exposure-specific molecular signature

In toxicogenomics, each significant variation observed between exposed and unexposed samples is attributed to the test compound and is commonly defined as its MOA. In the absence of cell death, the biological systems reach a new homeostasis, which can differ from the pre-exposure state. Hence, we first performed differential expression analysis for each dataset in the ENM collection and computed their pairwise similarity. Our results indicate that transcriptomic signatures are substantially dissimilar from each other (Supplementary Fig. 1), corroborating the hypothesis that transcriptional MOAs do not reveal common patterns of response to exposures^11^. These results are not surprising when considering the diversity of the experimental conditions represented in our collection.

Despite the intrinsic complexity of the individual datasets, we hypothesized that a signature of molecular alteration across ENM exposures exists and can be retrieved through meta-analysis. For this, we utilized an ensemble meta-analytical approach, prioritizing different aspects of the gene expression alteration, to highlight robust patterns of molecular deregulation detected in multiple ENM exposure systems in vivo and in vitro. Initially, we investigated the effect of including the nominal fold change derived from each study. Given the high similarity of the final ENM molecular signature (correlation > 0.75), we performed a canonical meta-analysis based solely on gene expression statistics (Supplementary Fig. 3) to rank 3,676 genes across the datasets (Supplementary Table 1).

**Fig. 2:**
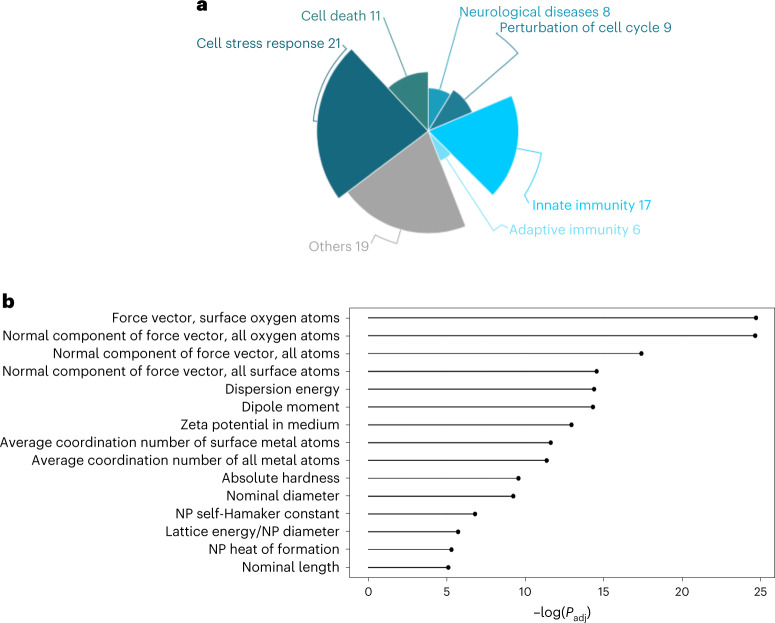
Characterization of the ENM molecular signature. **a**, Pie chart representing the functions associated with the meta-analysis rankings, grouped into six main biological categories (otherwise listed as ‘Others’). The number of pathways falling within each category determines their size. The complete list of pathways is shown in Supplementary Table 2. **b**, Molecular descriptors correlated with genes at the top of the ENM ranking. For each molecular descriptor, the top 10% of genes (*n* = 367 for each bar) in the dataspace, whose expression is correlated, are selected. A GSEA approach is used to highlight the descriptors whose correlated genes are enriched at the top of the ENM rank. The *P* value from the GSEA was corrected for multiple comparisons with the FDR method (*P* < 0.01). For more details, see Methods. NP, nanoparticle; *P*_adj_, adjusted *P* value.

The ENM signature has enriched biological pathways belonging to six main categories (cell stress response, innate immunity, cell death, cell cycle perturbation, neurological diseases and adaptive immunity) (Fig. 2a and Supplementary Table 2). Oxidative stress is a prevalent mechanism of ENM toxicity, as their reactive surface can induce hydroxyl radicals, which in turn results in lipid peroxidation, protein interference and DNA damage^12–14^. Cytotoxicity is modulated by ENM intrinsic characteristics^15^. Moreover, many ENMs have been previously associated with neuronal toxicity^2^, protein unfolding and fibrillation^16^, supporting our results.

**Fig. 3:**
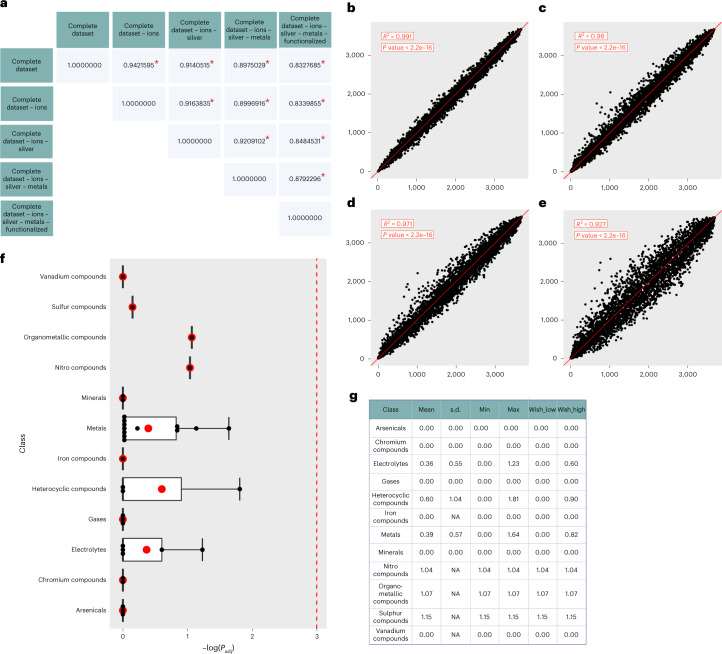
The ENM molecular signature is not shared with inorganic compounds. **a**, Correlation values between the meta-analysis rank obtained from the complete dataset and the rank obtained from partial datasets, progressively removing ion-releasing ENMs. Statistical significance between ranks has been determined with Kendall’s *τ* correlation test (marked with a red asterisk; *P* < 1 × 10^−16^). **b**–**e**, Comparison between the positions of the rank obtained by including the complete dataset and progressively removing ENMs potentially releasing ions. X-axis represents the gene positions in the original rank. The four plots include the following individual comparisons: complete set minus ions versus complete set (y-axis represents the gene positions in the rank minus ions) (**b**); complete set minus ions and silver versus complete set (y-axis represents the gene positions in the rank minus ions and silver) (**c**); complete set minus ions, silver and pristine metals versus complete set (y-axis represents the gene positions in the rank minus ions, silver and pristine metals) (**d**); and complete set minus ions, silver, pristine metals and functionalized metals versus complete set (y-axis represents the gene positions in the rank minus ions, silver, pristine metals and functionalized metals) (**e**). The fit is reported with a red line. In the top left boxes, the *R*^2^ and the *P* value of the limma fit are included. The *P* value was determined with a moderated *t*-test. **f**, Box plot of the gene set enrichment *P* values of the gene signatures of inorganic compounds derived from the CTD and the ENM molecular signature. The complete results are reported in Supplementary Table 2. The results are presented per compound category, as described by the CTD annotation. The significance threshold is indicated with a red dashed line. As the *P* value is expressed as minus logarithm, the significance threshold of 0.05 has been drawn at 2.995732. Individual data points are reported as black dots. The data are presented as mean values (red dots) ± standard error of independent experiments (*n* = 1, 1, 1, 1, 2, 12, 1, 3, 3, 5, 2, 3 for each bar, respectively). **g**, Box plot in **f** has been defined in terms of minima, maxima, bounds of box, whiskers and percentile.

Pathways related to both innate and adaptive immunity were strongly enriched (Supplementary Table 2). Interferons are known to mediate early innate and ancestral defence mechanisms, especially upon viral infection^17,18^, and have also been reported as being activated by exposure to some ENMs^19–22^. It is noteworthy that, in response to ENMs with diverse intrinsic properties, immune-related pathways are frequently altered across multiple cells and tissues, even those with no primary immune function. Toxicogenomics also concerns molecular response differences across exposures; therefore, we reported the ENM responses with specific physico-chemical characteristics (Supplementary Fig. 4). We next investigated the possible mechanisms of toxicity associated with this gene rank and tested whether ion release can explain the response of different biological systems to ENMs. For this, we progressively removed ion-releasing materials from the initial collection and compared the effect on the ENM signature (Fig. 3a). Our results indicate that ion release cannot alone describe the toxicity mechanism associated with the complete set of ENMs (Fig. 3b–e). Furthermore, we assessed whether removing ion-releasing materials affects the functional profile of the ENMs signature. As expected, the main functional categories initially identified are preserved (Fig. 2a and Supplementary Fig. 5). Altogether, these results may be relevant in future studies focusing on ENM ion release.

We then investigated which ENM characteristics are associated with our signature. We curated a set of 159 molecular descriptors and 46 experimental labels and computed their correlation with gene expression (Supplementary Table 3).

The top correlated descriptors suggest that nanoparticle thermodynamic stability and ion release has a role in defining toxicity (Fig. 2b). However, our results indicate that the nanoparticle-induced ion release is not overlapping with bulk ionic compounds. Interestingly, Gupta et al. hypothesized that the difference in the MOA of copper ionophores and nano-copper may relate to massive internalization of nanoparticles and their ability to trigger protein aggregation and proteasomal inhibition in lysosomes^23^. Indeed, we cannot exclude that intracellular localization and other unique properties of ENMs may induce different responses and, ultimately, differences in nucleus translocation. Enriched descriptors relevant for ENM–bio interactions inform on the tendency of the nanoparticles to aggregate, form protein corona and interact with cell membranes, eventually modulating the toxicological potential of nanomaterials. It is noteworthy that nanoparticle characteristics highlighted so far refer to their chemical composition and do not directly depend on particle size. Our analysis, however, also indicates the importance of geometrical descriptors. This suggests that nanoscale properties are relevant for toxicity in addition to the intrinsic size-independent representation of ENMs.

Finally, we investigated whether our gene signature is ENM specific or related more generally to xenobiotic exposure. To this end, we tested its similarity with other gene signatures associated with small molecules and inorganic compounds in their bulk form. We applied the same meta-analysis pipeline to the Open Toxicogenomics Project-Genomics Assisted Toxicity Evaluation System dataset, where the rat liver transcriptome is measured after multi-dose treatment with 158 small molecules. Our analysis showed substantial differences between small molecules and ENMs (Supplementary Fig. 6), suggesting that the identified transcriptional signature is indeed specific to ENMs. As the ENM molecular signature may still be shared with other types of compound, we tested whether it shares similarities with ionic and/or covalent compounds of the same elemental composition. We retrieved the gene signatures of 142 inorganic substances from the Comparative Toxicogenomics Database (CTD) and demonstrated that no bulk compound elicits significantly similar molecular responses to those observed upon ENM exposure (Fig. 3f and Supplementary Table 4).

This suggests that the unique properties of nanoforms influence the response of biological systems. Indeed, ENMs differ from drugs and inorganic compounds because of their unique physicochemical properties, requiring more information to be described than just their structure^24,25^.

## Identification of a regulatory model for ENM exposure

We hypothesized that the transcriptomics meta-analysis allows the identification of specific patterns of alterations common to long-term ENM exposure, in vitro and in vivo. We focused on the top-ranked 1,872 genes with the most significant functional enrichment (Methods).

When toxic doses are used, both in vitro and in vivo assays succeed in capturing ENM acute toxicity. However, these effects are less evident at sub-toxic doses, especially long-term. About 81% of the studies in our collection screened sub-toxic exposures; hence, we investigated the longer experimental time points and hypothesized that they could inform on shared responses of biological systems.

**Fig. 4:**
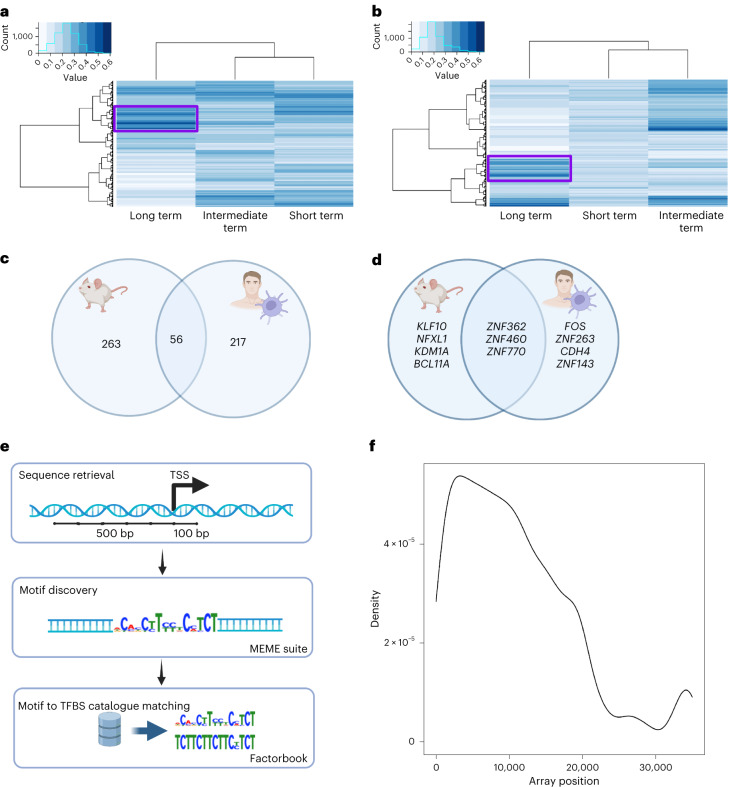
Identification of a gene regulatory model for ENMs exposures. **a**,**b**, Genes prioritized through functional analysis in vitro (**a**) and in vivo (**b**) are clustered according to the deregulation frequency following short, intermediate and long-term post-exposure monitoring. The cluster containing genes altered in at least 40% of the long-term monitoring samples (highlighted by the purple boxes) are selected for further investigation. **c**, Venn diagram showing the overlap between the two clusters of genes (in vitro and in vivo). **d**, Venn diagram showing the overlap of the regulatory motifs whose expression was statistically significant in the promoter regions of the two clusters of genes. **e**, Conceptual pipeline of the promoter analysis performed in this study. First, the DNA sequence of the [−500, +100] region around the TSS is retrieved. Motif discovery is performed through the MEME suite, finding all the DNA motifs between 6 and 15 base pairs that would satisfy a *P* value threshold of 0.05. MEME estimates the significance using an approximation to the *E* value of the information content of the motif. Finally, all the motifs are matched to the closest TFBS returned by the Factorbook database. **f**, Density plot of the position of genes regulated by the C_2_H_2_–ZNF family of transcription factors in the original complete ENMs dataset. On the *x* axis, the positions of genes ordered by the *P* value of their alteration is indicated. The *P* value of the gene expression is determined with a moderated *t*-test in the limma package R. On the *y* axis, the frequency of C_2_H_2_–ZNF targets for each position.

When comparing patterns of gene expression alteration in long-term post-exposure monitoring in vitro and in vivo (compare with Methods; Fig. 4a,b), we identified two distinct clusters of genes deregulated in at least 40% of instances (Fig. 4c and Supplementary Tables 5 and 6). In vivo and in vitro clusters contained 319 and 273 genes, respectively, sharing 56 genes (Supplementary Table 7). Functional annotation highlighted unfolded protein response (UPR), apoptosis, and alteration of cellular metabolism and cell membranes. UPR is induced after accumulation of misfolded proteins in the endoplasmic reticulum, activating processes to ease reticulum stress and ultimately inducing apoptosis if it cannot be reverted^26^. ENMs induce endoplasmic reticulum stress through various mechanisms, including reactive oxygen species, leading to UPR^27,28^. ENMs are also known to cause protein unfolding and fibrillation, potentially disrupting cellular proteostasis^16^. Complement proteins, acute phase and tissue leakage proteins form ENM coronas acting as ‘thorns’ inducing proinflammatory response^29^. All these responses are expected to occur in sub-toxic long-term exposures (Supplementary Table 8).

Long-term physiopathological changes are well captured by epigenetic modifications, while transient molecular responses are usually associated with transcriptional alteration^30^. Therefore, we investigated whether the long-term altered genes were commonly regulated.

We retrieved the promoter sequences of each gene both in vitro and in vivo and searched for conserved motifs around the transcription starting site (TSS; Fig. 4e). We identified ten DNA motifs in vivo and eight in vitro (*P* < 0.05) (Fig. 4d,e and Supplementary Table 9). It is noteworthy that, when the regulatory layer of the transcriptome is considered, the similarity between in vitro and in vivo increases from 20% gene similarity (Fig. 4c) to more than 40% regulatory motif similarity (Fig. 4d). The discovered motifs mainly bind members of the C_2_H_2_ zinc finger subfamily (C_2_H_2_–ZNF), eluding to their central role in the conserved response to ENMs^31^.

C_2_H_2_–ZNF are a large family of transcription factors in eukaryotes with key roles in development and differentiation^32^, the majority of which conserve binding sites and effectors throughout evolution^33^. They bind to repeated and contiguous motifs on target sites, often associated with transposable elements^33^. More than 700 C_2_H_2_–ZNF genes exist in humans, accounting for more than 2% of human genes^34^. Some members have also been linked to immunomodulation and inflammation^35–37^. Disruption of ZNF has been associated with metal-ion toxicity, a secondary effect of metal concentration changes, altering gene expression and DNA repair^38^. Importantly, while they have already been studied in plants as abiotic stress regulators^39^, their role in human toxicological responses remains unclear.

C_2_H_2_–ZNF transcription factors play a role in chromatin plasticity and recruitment of repressor complexes^40^. Modulation of chromatin structure is a commonly observed response to exogenous stimuli, for example, reduction of chromatin accessibility to avoid viral genomic insertion^41^. However, epigenetic mechanisms are involved in generic stress responses and can alter chromatin structure to produce transient translocation and nuclear reorganization^42,43^. This provides epigenetic memory of the environmental stimulus^44^. Notably, this mechanism has been observed in plants and simpler eukaryotic organisms^42,43^. Furthermore, topological associated domain borders are enriched in the C_2_H_2_–ZNF member CTCF (CCCTC-binding factor), whose binding determines chromatin domains^45^. Our results suggest that the epigenetic mechanisms found here, although still largely unexplored, can substantially aid the reconstruction of the ENM MOA. To validate our results, we selected ZNF362 (Fig. 3d), as it is expressed in all the main lung-derived cell lines (Supplementary Fig. 7a) and its epigenetic modifications have been associated with lung function in previous studies^46^. We performed dual luciferase assay to determine the activity of ZNF362 promoter in BEAS-2B cell line upon exposure to two different concentrations of carbon materials (MWCNT NM401 and carbon black) after 24 and 48 h, respectively (Supplementary Fig. 7c,d). At 24 h, carbon black induces ZNF362 especially at higher concentration (Supplementary Fig. 7c). At 48 h, ZNF362 is induced at high concentrations for NM401, although not significantly (Supplementary Fig. 7d). This is not surprising given the transient nature of the reporter vector transfections, which may be sub-optimal after 48 h. These findings provide an initial proof of concept of our model.

## C_2_H_2_–ZNF regulate toxicologically relevant genes

We hypothesized that the C_2_H_2_–ZNF model can be used to interpret a larger proportion of ENM transcriptomic responses, not limited to the 3,676 genes common across the studies analysed here. We investigated whether the most altered genes in response to individual ENMs are regulated by C_2_H_2_–ZNF in 84 transcriptomic datasets, including 19 not present in the initial meta-analysis (Supplementary Table 8). We show that the C_2_H_2_–ZNF regulatory model explains the most relevant transcriptional alterations in all individual transcriptomes (Fig. 4f and Supplementary Fig. 8).

Next, we evaluated whether the C_2_H_2_–ZNF model is relevant for ENM risk assessment.

A main theme in modern toxicology is the ability to generate exposure mechanistic models. Adverse outcome pathways (AOPs) emerged as robust multi-scale models linking chemical exposures to adverse outcomes. The annotation of toxicogenomics data into AOPs is currently evaluated by the Organisation for Economic Cooperation and Development to include toxicogenomic evidence in regulatory safety assessment^47^.

Here, we investigated molecular events regulated by C_2_H_2_–ZNF by enriching a recently curated gene annotation of AOPs^48^. Given the large number of genes targeted by any C_2_H_2_–ZNF, we restricted the analysis to specific C_2_H_2_–ZNFs identified in this study (Fig. 4d).

Events in AOPs are defined as molecular initiating events (MIEs), key events and adverse outcomes, based on their relationship with a specific endpoint. For this, we investigated the statistically significant over-representation of C_2_H_2_–ZNF targets against AOP-, MIEs-, key-events-, and adverse-outcomes-specific gene sets.

**Fig. 5:**
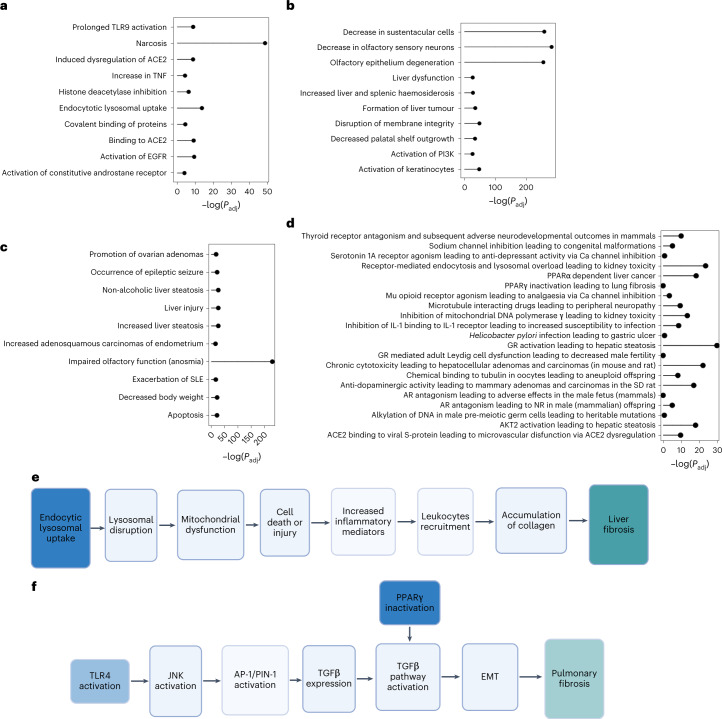
C_2_H_2_ family members enrich relevant AOPs. **a**–**c**, Top ten enriched MIEs, key events and adverse outcomes, with their respective adjusted *P* value (expressed as minus the logarithm of the value). TLR, toll-like receptor; ACE2, angiotensin-converting enzyme 2; TNF, tumour necrosis factor; EGFR, epidermal growth factor receptor; SLE, systemic lupus erythematosus; PPAR, peroxisome proliferator-activated receptor; IL-1, interleukin-1; GR, glucocorticoid receptor; SD, Sprague–Dawley; AR, androgen receptor; NR, nipple retention.; AP-1, activator protein 1; PIN-1, peptidylprolyl cis/trans isomerase NIMA-interacting 1; EMT epithelial mesenchymal transition. **d**, Top 20 enriched AOPs; JNK, jun N-terminal kinase; TGF, transforming growth factor. Besides the classical enrichment, we filtered pathways according to the proportion of individual key events enriched and discarded the ones in which less than one-third of the pathway is covered. **e**, Example of the ‘endocytic lysosomal uptake leading to liver fibrosis’ AOP, enriched by the regulatory model we identified. MIEs and adverse outcomes are represented in blue and green, respectively. Events reported as transparent do not pass the significance threshold (*P* < 0.05). **f**, Example of the ‘Toll-like receptor 4 activation and peroxisome proliferator-activated receptor gamma inactivation leading to pulmonary fibrosis’ AOP, enriched by the regulatory model we identified. Graphical annotation is the same as in **e**. The statistical significance in **a**–**e** was determined with a Fisher test, and multiple comparisons adjustment method was performed with the Bonferroni correction.

The enrichment of narcosis (*P* < 7.82 × 10^−22^), interaction with membrane receptors (*P* < 1 × 10^−4^) and lysosomal dysfunction (*P* < 1.09 × 10^−6^) MIEs suggest that the C_2_H_2_–ZNF regulatory model captures the initial events of the ENM–host interaction and encompasses cellular homeostasis disruption (Fig. 5a).

Furthermore, activation of the phosphoinositide 3-kinase (PI3K) signalling pathway (*P* < 3.58 × 10^−12^) induces inflammation and reactive oxygen species production (*P* < 6.62 × 10^−11^; Fig. 5b). Among PI3K effectors, NFkB has a central role in ENM response (Supplementary Table 10). Finally, the previously discussed effect on membrane homeostasis and immune system is supported by adverse outcome enrichment (Fig. 5c).

We also evaluated the enrichment of AOPs, restricting cases in which less than one-third of key events associated with an AOP are significant. We report the 20 most significant enrichments and graphically describe two AOPs, showcasing that the C_2_H_2_–ZNF model is directly linked to ENM MOA (Fig. 5d–f).

Focusing on dose-dependent molecular alterations is also relevant for regulatory purposes^49^. Therefore, we evaluated the portion of dose-dependent genes that are regulated by C_2_H_2_–ZNFs in 62 studies^50^. We demonstrate that C_2_H_2_–ZNF regulates on average 55.3% of all dose-dependent genes across a wide range of experimental conditions (Supplementary Fig. 9).

Taken together, these results show that C_2_H_2_–ZNFs can explain molecular responses to ENMs also in a regulatory relevant perspective.

## The C_2_H_2_–ZNF model is conserved in eco-toxicological species

**Fig. 6:**
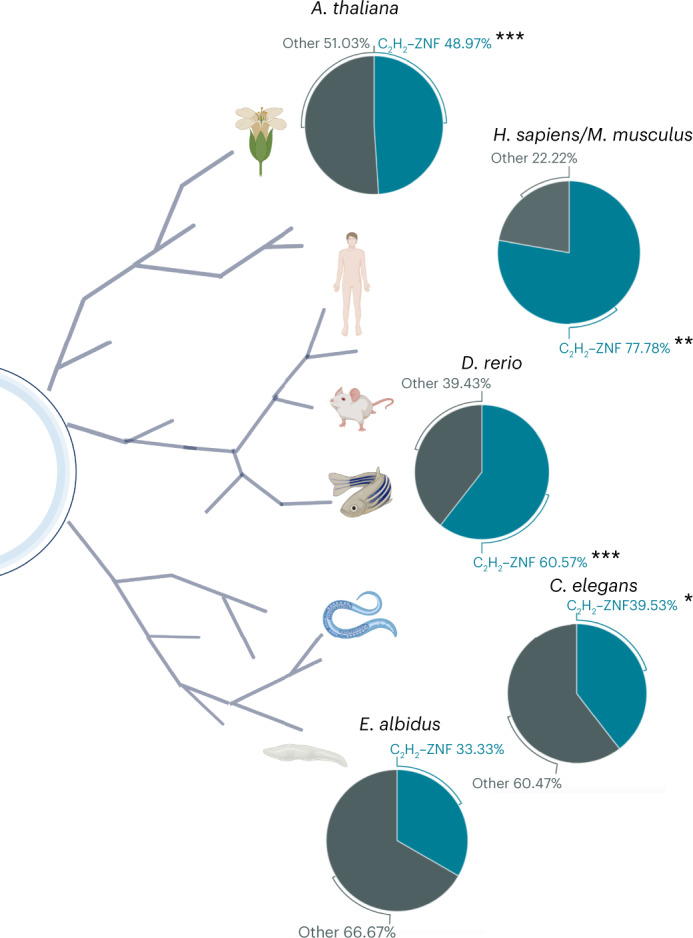
The C_2_H_2_ model is conserved in other non-mammal species. Overview of the species included in our study and relative proportion of C_2_H_2_–ZNF TFBS in the promoter region of the genes involved in their adaptation response. The statistical significance was estimated with the Fisher test and is indicated by the number of asterisks. *P < 0.005, **P = 1.4 × 10^−8^ and ***P < 2.2 × 10^−16^.

Finally, we hypothesized that C_2_H_2_–ZNFs mediate ENM transcriptomic responses in other species of eco-toxicological interest. We analysed 17 datasets recently curated, including ENM exposures to *Danio rerio*, *Caenorhabditis elegans*, *Enchytraeus* *albidus* and *Arabidopsis thaliana*^11,51^ (Supplementary Table 11). Our results indicate that in non-mammal organisms the ENM response is also regulated by C_2_H_2_–ZNF (Supplementary Table 12). Interestingly, the relative proportion of C_2_H_2_–ZNF transcription factor binding sites (TFBS) decreases across the phylogenetic tree, suggesting a possible association with organismal complexity^34^ (Fig. 6). The organisms considered here have variable levels of organization of adaptive and innate immunity. Plants show host immunity controlled by polymorphic host genes, where resistance protein-mediated defence is based on ‘altered-self’ mechanisms of recognition^52^. These mechanisms commonly use epigenetic modification and chromatin remodelling to establish infection memory, achieving immunity even in the absence of specialized immune cells^53^. Recently, ref. ^54^ described immune-deregulation and stress response as a shared feature to lithium cobalt oxide exposure across taxonomic groups.

## Conclusions

Response to environmental stimuli is a primitive function of living organisms that influences their evolution through epigenetic mechanisms that minimize harmful events, while increasing their fitness. Although ENMs originated during industrialization, all organisms were exposed to natural nanoparticles across geological eras^55–57^. This suggests that responses and mechanisms of adaptation result from a long evolutionary exposure history to nano-sized matter. Our results suggest that a very ancestral regulatory mechanism may be conserved across the tree of life and explain the response to ENMs. This proposed model captures commonalities of molecular response beyond the divergence of individual studies and points to shared immunomodulation of ENMs, even in non-immune cells.

The stability of the regulatory layer across species paves the way to new toxicological tests that can bridge ecotoxicology and human toxicology^58^, possibly facilitating the development of more effective in vitro–in vivo extrapolation.

Exposome impacts on the epigenome, contributing to disease susceptibility^59^. Hence, association between DNA modifications (for example, DNA methylation) and exposures to environmental factors have been widely investigated^60,61^. Moreover, the epigenome regulates chronic responses to drugs^62^. Our results further suggest the role of additional layers of (epi)genomic regulation on long-term responses to ENMs.

However, efforts such as the one described here are challenged by limited homogeneity in reporting nanosafety experiments^63,64^. Nonetheless, our model is statistically robust and validated in the largest collections of curated nanotoxicogenomics data. Moreover, it is potentially relevant for risk assessment in multiple species and hence throughout the ENMs life cycle.

In conclusion, this study suggests that a wide range of biological systems respond to ENMs through a set of commonly regulated genes. We demonstrate that this signature is specific to ENMs and interlinked to their nano-properties. This study sheds light on an unexplored layer of ENM MOA and proposes a solution to the ‘one-chemical-one-signature problem’, currently limiting the use of toxicogenomics in chemical safety assessment.

## Methods

### Data collection and pre-processing of nanomaterial datasets

The meta-analysis of ENMs toxicogenomics studies in vitro and in vivo can identify common molecular MOA independent from the biological system under evaluation. To this end, we implemented a meta-analysis of 66 transcriptomic datasets derived from the public data collection curated by ref. ^8^ (https://zenodo.org/record/3949890#.YlPUri0RqH0), supplemented with data previously published in ref. ^10^ (GSE157266) and ref. ^9^ (GSE148705) (Supplementary Table 8). From the original collection, we excluded rats’ datasets and the ones based on old microarray platforms, as they shared little probes with the more recent versions (Supplementary Table 8).

The datasets GSE148705 and GSE157266 were pre-processed using the eUTOPIA software (version commit December 2021), as previously described^8,65^. Briefly, we filtered probes with a value higher than the 0.8 quantile against the negative control in at least 75% of the samples. Data were normalized between arrays using quantile normalization^66^. No batch correction was needed for the dataset GSE148705, while GSE157266 was corrected for technical variation associated with variables ‘dye’ and ‘slide’ using the ComBat method^67^. Finally, we used the limma package (version 3.52.4) to compute the gene expression difference between each exposure in the dataset and the corresponding controls, correcting the *P* value using the Benjamini–Hochberg procedure. The aggregated, normalized and corrected expression matrix was then exported with no additional filtering.

In this study, we selected all pairwise comparisons between time–dose exposures and their respective controls. The final dataset comprised 584 specific exposure conditions (treatment, exposure time and dose, and biological system) and 3,676 genes, ranging across various human and mouse tissues and cell types.

The pool of 3,676 genes represents the intersection of genes present in all the experiments, limiting the mouse–human conversion to 1:1 orthology relationships (that is, where both genes in the pair have only one ortholog in the other species). The ortholog genes were converted using the getLDS function of the biomaRt R package (version 2.52.0)^68^. All data are available in the online Zenodo repository (10.5281/zenodo.7674574).

#### Collection and pre-processing of transcriptomics data for drug exposure and chemical compounds

To assess the specificity of the ENM signature, raw microarray data for 158 drugs was downloaded from the Open Toxicogenomics Project-Genomics Assisted Toxicity Evaluation System database^69^. We applied the same pipeline to the in vivo exposures of rats to three dose levels of each drug.

Raw data were imported into R using the justRMA function from the R library Affy (version 1.60.0)^70^. Probe annotation to Ensembl genes was performed by the custom annotation files rat2302rnensgcdf (v. 22.0.0), downloaded from the brain array website, leading to 12,153 genes. The expression values were quantile normalized by means of the normalizeQuantile function from the R limma library (version 3.52.4)^66^. Differential expression analysis was performed for each drug, for each combination of dose level and time point (1,839 comparisons). The analyses were performed comparing the treated samples to the matched control samples of the same time point. As a result, log_2_ fold changes, *P* values and adjusted *P* values (by means of the false discovery rate (FDR) correction) were retrieved for all genes for each comparison.

To make a comparable evaluation, we only selected genes that had been included in the meta-analysis rank. As for chemical compounds, we downloaded, from the CTD, 142 gene signatures of ionic and covalent compounds^71^. We discarded gene sets smaller than 15 and bigger than 1,000, as it would have altered the statistics of the test, as well as hydrocarbons, alcohols and ethers, as they are classified as organic. For each of the remaining gene sets, we performed a gene set enrichment analysis (GSEA) of the ENM-associated rank and considered a significance threshold of *P* value adjusted to 0.05.

#### Collection of ecotoxicological transcriptomics data

To test the translatability of our model to non-mammal species of eco-toxicological interest, we verified whether genes altered in response to ENM in other non-mammal species are regulated by C_2_H_2_–ZNF transcription factors. To this aim, we downloaded from the Gene Expression Omnibus (GEO) seven datasets covering 17 exposures to well-known eco-toxicological model organisms (*D. rerio*, *C. elegans*, *E. albidus* and *A. thaliana*) and report the lists of differentially expressed genes (GSE80461, GSE32521, GSE70509, GSE73427, GSE77148, GSE41333, GSE47662)^11,51^ (Supplementary Table 11). The genes have been used to perform promoter analysis as previously described for the discovery collection (compare with ‘Promoter analysis’).

### Characterization of the 584 experimental conditions

From the initial collection, we identified 584 experimental conditions that are unique because of the biological system, ENM or experimental condition used. We manually annotated each experimental condition by curating the information in the original publications (Supplementary Table 3). First, we grouped samples according to the biological system and exposure setting. We also included the exposure duration by grouping samples into short, intermediate and long exposures. Different thresholds were defined for in vivo and in vitro. In detail, for in vitro experiments, exposures have been considered short at 24 h, intermediate between 24 and 72 h and long after 72 h, respectively, as we expected the (sub-)acute toxicity to be observed within the first days of the exposure. Indeed, ref. ^72^ recently reported that in vitro systems tested between 6 and 72 h reproduce a scenario accounting for acute toxicity assessment of chemicals.

For in vivo exposures, thresholds were 3 days (short), between 3 days and 1 month (intermediate) and more than 1 month (long-term). In the health chemical evaluation, the 28 day exposure Organisation for Economic Cooperation and Development protocols are considered as the preliminary tests to assess long-term toxicity, while chronic toxicity studies should have a length of 12 months^73–76^ (Supplementary Fig. 10).

As for doses, only nominal doses for each experiment were available. It is noteworthy that nominal doses are not comparable between the exposures, further complicated by the heterogeneity of the measuring units reported (Supplementary Fig. 2b). We investigated that the majority of the studies included here tested sub-toxic dose exposures via a semi-automatic pipeline to scan the original manuscripts. We retrieved all the PubMed Central identifiers of the original articles. When the experimental details were not available, we considered the cited protocol in its references.

We used the BioPython Entrez (version 1.81)^77^ api to retrieve the documents through the PubMed Central IDs. Finally, we parsed the resulting XML documents and searched the abstract and/or the whole article for keywords (Supplementary Table 8) referring to sub-toxic doses. Each positive result was returned with its context and manually validated. For a reduced number of datasets, the corresponding articles could not be retrieved automatically, so they were manually checked. We were able to find indications of sub-toxic doses in 47 out of 58 publications.

The nanomaterials used in the experiment were classified according to the chemical characteristics and the presence or absence of functionalized groups.

A panel of information was extracted from the original publications (when possible), covering crystal phase, purity, absence of endotoxins, coating, stabilizer and supplier information, as well as protocol information.

Finally, when characterized, data were reported regarding the nominal diameter, length and specific surface area; transmission electron microscopy diameter, width and length; Brunauer-Emmett-Teller surface area; number of walls; dynamic light scattering mean diameter and polydispersity index in water and medium; and zeta potential in water and medium.

### Computation of molecular descriptors

A set of 159 ENM descriptors covering both molecular and electronic structure properties was computed. Liquid drop model molecular attributes^78^ are calculated assuming that ENM can be represented as a spherical drop, where elementary molecules are tightly packed, while the density of clusters is equal to the particle mass density^78,79^. We computed the Wigner–Seitz radius (*r*_w_), the number of ENMs in the analysed agglomerate (*n*), the number of surface elements (*S*), the surface–volume ratio (*SV*) and the aggregation parameter (*AP*)^78^. The Wigner–Seitz radius characterizes the minimum radius of interactions between individual molecules and is represented by equation (1):

1 $$r_{\mathrm{w}} = \left( {\frac{{3M}}{{4\pi \rho N_{\mathrm{A}}}}} \right)^{\frac{1}{3}}$$

where *M* is the molecular weight, *ρ* is mass density, and *N*_A_ is the Avogadro constant.

The number of ENMs in the agglomerate (*n*) is represented using equation (2):

2 $$n = \left( {\frac{{r_0}}{r_{\mathrm{w}}}} \right)^3$$

where *r*_0_ is the radius of each ENM.

The number of surface elements (*S*) is represented by equation (3):

3 $$S = 4n^{ - \frac{1}{3}}$$

where *S* describes the ratio of surface molecules to molecules in the volume (or surface ENMs in agglomerates).

The surface–volume ratio (*SV*) is represented using equation (4):

4 $$SV = \frac{S}{{1 - S}}$$

where *SV* is the feature that describes the ratio of surface molecules to molecules in volume (or surface ENMs in agglomerates).

Size-dependent interfacial thickness (*h*) was calculated with equation (5)

5 $$h = 0.01 \times (T - 273) \times r^{0.35}$$

where *r* is the nominal size of the ENM and *T* is temperature^80^.

The ENM electronic structure descriptors were computed by density functional theory and semi-empirical quantum chemical methods, while the Hamaker constants were evaluated from atomistic force fields and a continuum method^81,82^. ENMs interact via long-range van der Waals interaction, which is a major contribution to calculating the adsorption energies of biomolecules in water. Therefore, Hamaker constants are evaluated to describe bio–nano interactions in water through an atomistic force field approach and via Lifschitz theory^82^. In the Lifschitz theory^82^ two materials are interacting through a medium; the Hamaker constant for the ENM and a biomolecule in water is calculated from optical parameters that are experimentally determined (Supplementary Table 13), while in the force field approach long-range dispersion interaction is calculated using the Lorentz–Berthelot rules for sigma (atom size) and epsilon (atom–atom interaction amplitude)^83,84^. For metal ENMs, we used CHARMM force field parameters^85^. For metal oxides and carbon ENMs as well as amino acids, lipids and sugars, the force fields are reported in ref. ^86^. Considering all atom–atom interactions between two molecular entities, the Hamaker constant is derived by an approximation of the combined sigma and epsilon dispersion parameters^87^. In this work, we also considered the interaction between two ENM pieces in water. The geometric structures of the bulk ENMs were optimized with density functional theory and the Perdew-Burke-Ernzerhof functional^88^ using the SIESTA code^89^. The band gaps were also calculated by PBE^88^, while the heat of formation, electronegativity, absolute hardness, dispersion energy per atom, dipole moment and static polarizability descriptors^81^ were obtained on the self-consistent field level through the semi-empirical code MOPAC (http://OpenMOPAC.net) using the PM6-D3^90^ parametrization. Finally, ionization potentials, electron affinities, and the global electrophilicity index were computed through self-consistent charge calculations (ΔSCC calculation) for the electronic states of the neutral and ion ENMs via the GFN1-xTB parameterization of the GFN-xTB code^91–95^.

We further included a set of atomistic descriptors that are based on the chemical composition, potential energy, lattice energy, topology, size and force vectors^96,97^. Constitutional descriptors are the counts of atoms of different identity and/or location. Potential energy descriptors are derived from the force-field calculations, corresponding to the arithmetic means of the potential energies for specific atom types and/or locations in the ENM. Lattice energies are based on the same potential energies but presented as per metal oxide nominal units (MxOy) and describe the energy needed to rip away said unit from the ENM surface. The coordination number of atoms is defined as the count of the neighbouring atoms that lie inside the radius,

6 $$R = 1.2 \times (R_{\mathrm{M}}\;{\mathrm{and}}\;R_{\mathrm{O}})$$

where *R*_M_ and *R*_O_ are the ionic radii of metal and oxygen ions, respectively. A low coordination number indicates that some atoms have missing neighbours and thus makes the ENM more unstable. The size was derived from the actual calculated ENM diameter. The force vector lengths have been derived from the structure optimization. For example, to derive the average length (*V*) of the surface normal component of the force vector for a shell region atom, its coordinates (*x*, *y*, *z*), force vector components (*f*_*x*_*, f*_*y*_*, f*_*z*_) and distance from the centre of mass (*d*) are used:

7 $$V = \frac{{(xf_x + yf_y + zf_z)}}{d}$$

Sample values for TiO_2_ (10 nm) are reported (Supplementary Table 14). For amorphous ENMs a multi-step procedure was used requiring the simulation of bulk metal or metal oxide materials above their melting temperature, the extraction of ENMs with the desired size and shape, and their subsequent cooling at the temperature of interest with a prescribed rate. Such a procedure has been applied to build spherical amorphous ENMs with the aid of the automated Enalos Demokritos KNIME nodes. All the data are hosted at the NanoPharos database (db.nanopharos.eu) and were converted into a ready-for-modelling format.

### Meta-analysis implementation

We implemented a consensus of three algorithms for meta-analysis to prioritize the shared 3,676 genes.

As previously proposed^98,99^, our pipeline is based on the effect-size, *P* value-based and rank-product methods. Usually, meta-analysis frameworks are based on effect-size methods, assessing within- and between-study variations across multiple studies. These methods outperform others when there is large between-study variation and small sample sizes. To implement it, the ‘effect_sizes’ function from the esc R package (version 0.5.1) was used with the *P*-value argument and the ‘chi_esc’ function^100^. The Fisher’s sum of logs method combines individual *P* values. Fisher’s sum of logs method was implemented by using the ‘sumlog’ function of the R package metap (version 1.8), giving as input the *P* values of each gene^101^. Finally, the rank product is a non-parametric statistical method to combine differential gene expression analysis results from individual studies based on the within-study gene ranks. To this end, the genes in each experiment were ranked based on the relevance of their associated *P* values, and the ‘RP.advance’ function of the RankProd R package (version 3.24.0) was used to merge them via one-class analysis of the rank-product method^102,103^. This function allows combining data coming from different studies, such as in the case of datasets generated by different laboratories. For each method, a rank was generated. Finally, all the ranks were combined through the Borda function of the TopKlists R package (version 1.0.8)^104^. The final mean rank is reported in Supplementary Table 1.

### GSEA and feature selection step

To select the most biologically relevant portion of the rank, we performed a GSEA of the meta-analysis rank on five databases (Wikipathways^105^, Gene Ontology^106^, Reactome^107^, Kyoto Encyclopedia of Genes and Genomes^108^ and MsigDB^109^). In each case, the ‘fgsea’ function from the fgsea R package (version 1.22.0) was used^110^. For each test, we identified the position of the rank having the highest peak value of cumulative enrichment statistics. We created a reduced representation of the meta-analysis gene rank by setting as a threshold the top 10th percentile of such values (1,873 genes).

### Computation of the frequency score and hierarchical clustering

To find genes associated with in vitro and in vivo long-term exposures for each gene of the reduced meta-analysis rank, we calculated a frequency score as the percentage of samples in which the gene was statistically significant.

The genes were clustered according to the Euclidean distance of their frequency scores. The hierarchical clustering algorithm, with Ward linkage method, implemented into the ‘hclust’ function of the R package ‘stats’ (version 4.2.0) was used.

For each type of exposure system, we selected the cluster with the most frequently deregulated genes. To functionally annotate them, we performed a pathway enrichment analysis through the EnrichR online tool (accessed in 2021), using the MsigDB and Reactome databases^107,109,111–113^.

### Computation of the molecular descriptors–gene expression correlation

To identify associations between ENM chemical properties and molecular alterations induced in cells and organisms by their exposure, the Pearson correlation coefficient was computed for each pair of gene and molecular descriptor, after a pre-processing step. In particular, a Winsorize function of the DescTools R package (version 0.99.43)^114^ was used to replace extreme values of log_2_ fold changes with less extreme ones. Moreover, a cube root transformation was applied to the molecular descriptor values.

As the molecular descriptor data layer contains missing data, the Pearson correlation was computed for the subset of samples where values were available.

For each descriptor the top 10% of the most correlated genes were selected. First, the gene sets were enriched against the Kyoto Encyclopedia of Genes and Genomes pathways by means of the FunMappOne tool (version commit December 2021)^115^. Only pathways with FDR-corrected *P* < 0.05 were considered significantly enriched. The molecular descriptors were further clustered in nine groups based on the Jaccard Index similarity of the shared enriched pathways. Lastly, the fgsea R package (version 1.22.0)^110^ was used to perform a GSEA analysis and identify the molecular descriptors whose set of associated genes is enriched on the top of the ranked list of genes identified with the meta-analysis approach. Only molecular descriptors with an adjusted *P* < 0.01 were selected.

#### Promoter analysis

For each gene in the subset of interest, the sequence of the promoter region [−500 bp, +100 bp] around the TSS was downloaded using the biomart package and the getSequence function in ‘coding_gene_flank’ mode^68^. In this modality the function returns the flanking region of the gene including the untranslated regions.

Motif discovery was conducted with the MEME software suite (version 5.5.1)^116^. The motif site distribution was set as any number of repetitions; the search was restricted to motifs ranging between 6 and 15 bases and the *P*-value threshold to 0.05.

For each result, the Factorbook database was interrogated to explore the TFBS (https://www.factorbook.org/). Factorbook is a transcription factor-centric web-based repository associated with ENCODE ChIP-seq data, as well as multiple databases of TFBSs. We selected the TFBS that best matches the query according to the tool.

For each organism (*Homo sapiens* and *Mus musculus* in the discovery set, and *D. rerio*, *C. elegans*, *E. albidus* and *A. thaliana* in the eco-toxicological comparison) we annotated whether the TFBS would be recognized by a C_2_H_2_–ZNF member. To evaluate the statistical significance of C_2_H_2_–ZNF overrepresentation, we performed a Fisher test with the fisher.test function in the stats R package. We used as a background the set of non-redundant transcription factor binding profiles provided in the JASPAR database^117^. The contingency matrix was built by using the set of TFBS of the C_2_H_2_–ZNF family members and all the others, respectively.

#### Dual luciferase assay

BEAS-2B cells (ATCC, CRL-9606) were grown in BEGM (Lonza, CC-3170). Cells were cultivated in 75 cm^2^ culture flasks at 37 °C with a humidified atmosphere of 5% CO_2_. For all experiments, 500 µl of cells was seeded at a density of 3.75 × 10^3^ cells per ml in 48-well plates. Cells were then left to rest overnight before transfections. One hour before transfection, the media was replaced with 225 µl of fresh BEGM per well. Two vectors were used for transfection: human cytomegalovirus (CMV) (positive vector control) and the ZNF362, created with VectorBuilder (Supplementary Fig. 7b). Per well, 0.25 µg of DNA vector in Opti MEM reduced serum medium (Gibco, 31985062) was added 1:1 with Lipofectamine 3000 reagent 6% V/V (ThermoFisher Scientific, 15338030) and P3000 enhancer reagent 4% V/V in Opti MEM reduced serum medium (Gibco, 31985062). About 25 µl of this transfection solution was added per well and mixed by gentle agitation of the plate. Twenty-four hours post-transfection, cells were exposed to one of the following: NM401 (JRC MWCNTs-NM401-JRCNM04001) or carbon black (CB, Orion Engineered Carbons, Printex 90) nanomaterials at either low (20 µg ml^−1^) or high (100 µg ml^−1^) concentration; or nefazodone hydrochloride (Sigma-Aldrich, N5536) at low (25 µM) or high (50 µM) concentration. NM401 and CB nanomaterials were prepared according to the nanogenotox protocol (https://safenano.re.kr/download.do?SEQ=175). Briefly, in a glass vial, 0.5% of final stock volume of ethanol was added to the initial weighed nanomaterial powder; 0.05%W/V BSA-BEGM was added for a final stock concentration of 0.2 mg ml^−1^. The vial was then sonicated 2 times for 15 min in a water bath; this stock solution was then diluted in 0.05%W/V BSA-BEGM to create final solutions, and final solutions were sonicated for 15 min before addition to the wells. Vehicle control (VC) for nanomaterials was 0.05%W/V BSA-BEGM. Nefazodone hydrochloride was prepared in 0.05%W/V BSA-BEGM and DMSO (final DMSO concentration in well of 0.5%). VC for nefazodone hydrochloride was 0.5% DMSO in 0.05%W/V BSA-BEGM. Exposures were performed for 24 and 48 h.

The Dual-Glo luciferase Assay System (Promega, E2920) was used as per the manufacturer’s guidelines to measure firefly and renilla luciferase activity, on a Spark multiplate reader (Tecan).

There were three samples measured for each vector and vehicle control, for each exposure. The mean signal of three background wells (cells only) was used to subtract background from luciferase measurements. The firefly luciferase activity was normalized to renilla luciferase and power transformed. When present, outliers were removed with the boxplot function in R. The *t*-test was used to investigate the differences between the experimental and control samples.

#### Dose-dependent gene analysis

To verify if the C_2_H_2_–ZNF model can explain the dose-dependent portion of the ENM response, a dose–response analysis of 62 studies derived from 33 datasets (all initially included in the analysis but GSE146708) was performed following the strategy implemented in the BMDx tool (version commit February 2022)^118^. Briefly, multiple models were fitted, and the optimal model was selected as the one with the lowest Akaike information criterion. The effective doses (BMD, BMDL and BMDU) were estimated under the assumption of constant variance. The benchmark response was identified by means of the standard deviation approach with a benchmark response factor (BMRF) of 1.349, corresponding to a minimum of 10% difference with respect to the controls. Only genes with lack-of-fit *P* > 0.01 and with estimated benchmark dose (BMD), benchmark dose lower confidence limit (BMDL) and benchmark dose upper confidence limit (BMDU) values were deemed relevant. Genes with BMD or BMDU values higher than the highest exposure dose were removed. Furthermore, genes whose ratio between the predicted doses is higher than the suggested values (BMD/BMDL > 20, BMDU/BMD > 20 and BMDU/BMDL > 40) were removed from the analysis.

#### AOP enrichment analysis

To enrich key events and AOPs, we exploited the recently curated annotation from ref. ^48^. For each gene set, we performed an enrichment of the C_2_H_2_–ZNF targets as derived from ref. ^119^. For individual events, the significance threshold was set at *P* value adjusted to 0.05.

As for the complete AOPs, we first enriched as described the genes annotated to each pathway and discarded *P* values higher than 0.05. In a next step, we evaluated if at least one-third of the events contained in it passed the same threshold of significance (0.05).

### Reporting summary

Further information on research design is available in the Nature Portfolio Reporting Summary linked to this article.

## Online content

Any methods, additional references, Nature Portfolio reporting summaries, source data, extended data, supplementary information, acknowledgements, peer review information; details of author contributions and competing interests; and statements of data and code availability are available at 10.1038/s41565-023-01393-4.

## Supplementary information

Supplementary Information Supplementary Figs. 1–10.

Reporting Summary

Supplementary Table 1 The file contains all the supplementary tables as a combined workbook with multiple tabs. The tabs have been named to match the individual tables. Each table has a legend or description of its content.

## Data Availability

The pre-processed version of the transcriptomic datasets included in the discovery datasets, that is, ENM exposures of human and mouse samples, have been previously deposited at https://zenodo.org/record/3949890#.YlPUri0RqH0. The original datasets can be accessed at Array Express (https://www.ebi.ac.uk/biostudies/arrayexpress) with the entry code EMTAB6396 and at GEO (https://www.ncbi.nlm.nih.gov/) under accession numbers GSE103101, GSE112780, GSE113088, GSE117056, GSE122197, GSE127773, GSE146708, GSE148705, GSE157266, GSE16727, GSE17676, GSE19487, GSE20692, GSE29042, GSE35193, GSE39330, GSE41041, GSE42066, GSE42067, GSE42068, GSE43515, GSE45322, GSE45598, GSE4567, GSE46998, GSE46999, GSE50176, GSE51186, GSE51417, GSE51421, GSE51636, GSE53700, GSE55286, GSE55349, GSE56324, GSE56325, GSE60797, GSE60798, GSE60799, GSE60800, GSE61366, GSE62253, GSE62769, GSE63552, GSE63806, GSE68036, GSE75429, GSE79766, GSE81564, GSE81565, GSE81566, GSE81567, GSE81568, GSE81569, GSE82062, GSE84982, GSE85711, GSE88786, GSE92563, GSE92900, GSE92987, GSE96720, GSE98236 and GSE99929. Transcriptomic datasets used for the eco-toxicological analysis are freely available at GEO under accession numbers GSE80461, GSE32521, GSE70509, GSE73427, GSE77148, GSE41333 and GSE47662. Transcriptomic datasets of small molecule exposure (Open-TG GATEs) have been downloaded from https://dbarchive.biosciencedbc.jp/en/open-tggates/download.html in November 2020. Functional data were downloaded from https://www.gsea-msigdb.org/gsea/msigdb/ version 7.2. All the other relevant data and data supporting the findings of this study have been deposited in the online Zenodo repository (10.5281/zenodo.7674574).

## Code availability

All the relevant and custom code supporting the findings of this study has been deposited in the online Zenodo repository (10.5281/zenodo.7674574) and on Github at https://github.com/fhaive/metanalysis_toxicogenomic_data.
