# Supplementary material for: An ancestral molecular response to nanomaterial particulates
Source: Nat Nanotechnol. 2023 May 8;18(8):957–66. doi: 10.1038/s41565-023-01393-4 (PMC10427433; doi:10.1038/s41565-023-01393-4)
Supplement: Supplementary file 1 — Supplementary Figs. 1–10. [file 41565_2023_1393_MOESM1_ESM.pdf]

nature nanotechnology

Analysis

https://doi.org/10.1038/s41565-023-01393-4

An ancestral molecular response to nanomaterial particulates

In the format provided by the authors and unedited

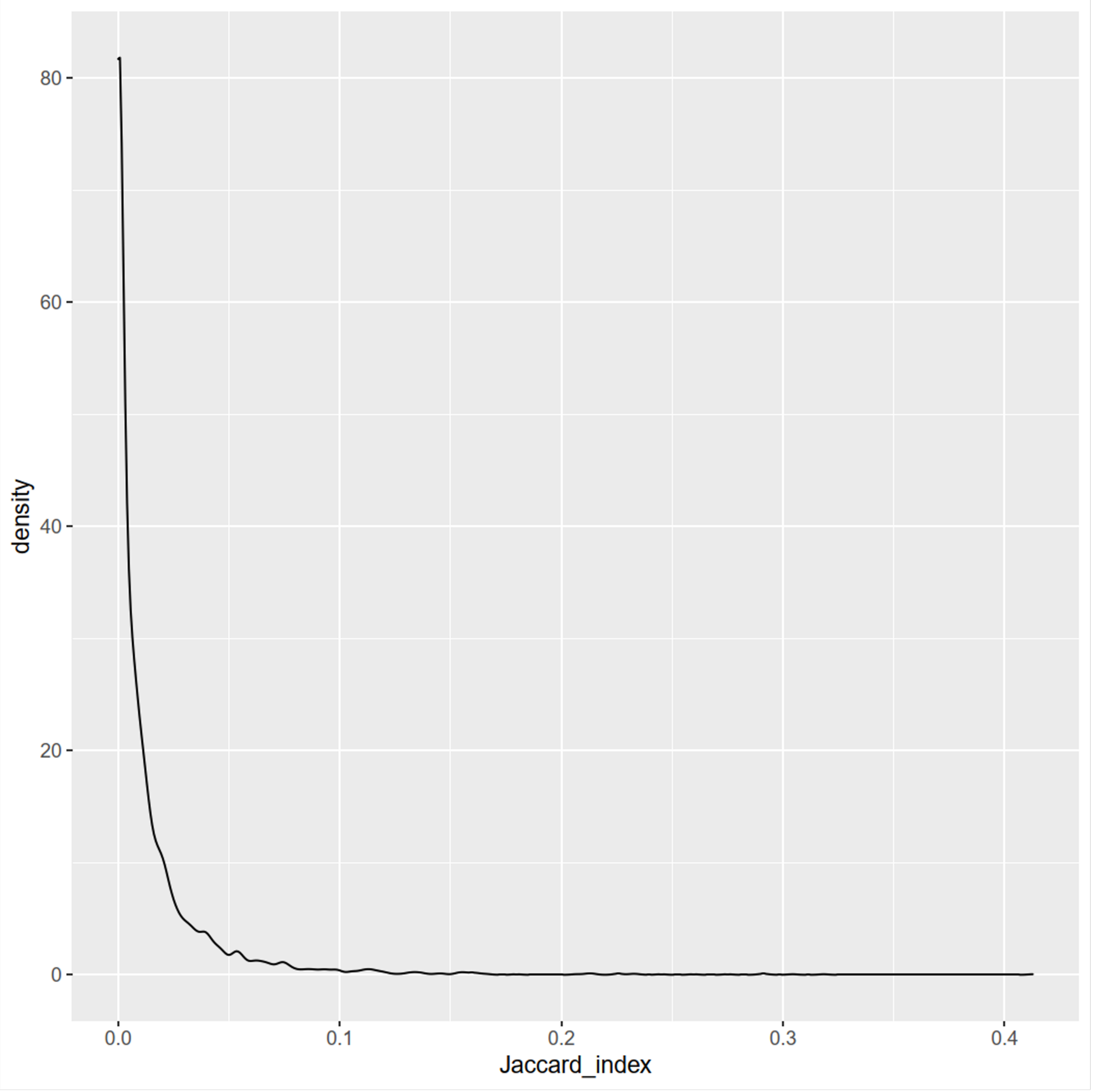

Figure S1: Distribution of the Jaccard index similarity values, between the differentially expressed gene lists derived from the datasets included in the collection.

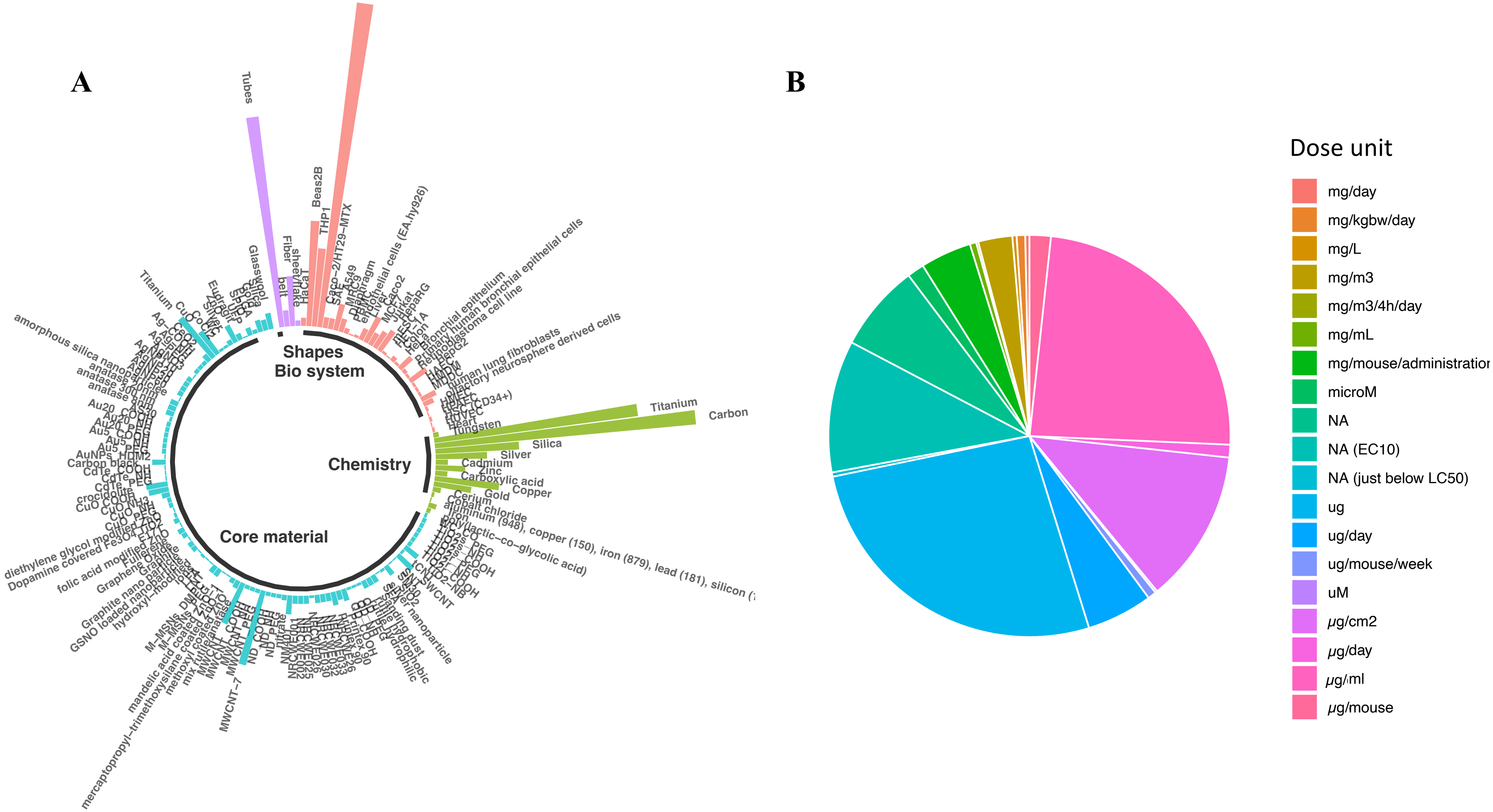

Supplementary Figure 2: A. Circular Barplot representing the main classes of ENMs and exposed systems in the analysis data collection. **B.** Pie chart of the dose units reported in the original publications of the data used in the analysis.

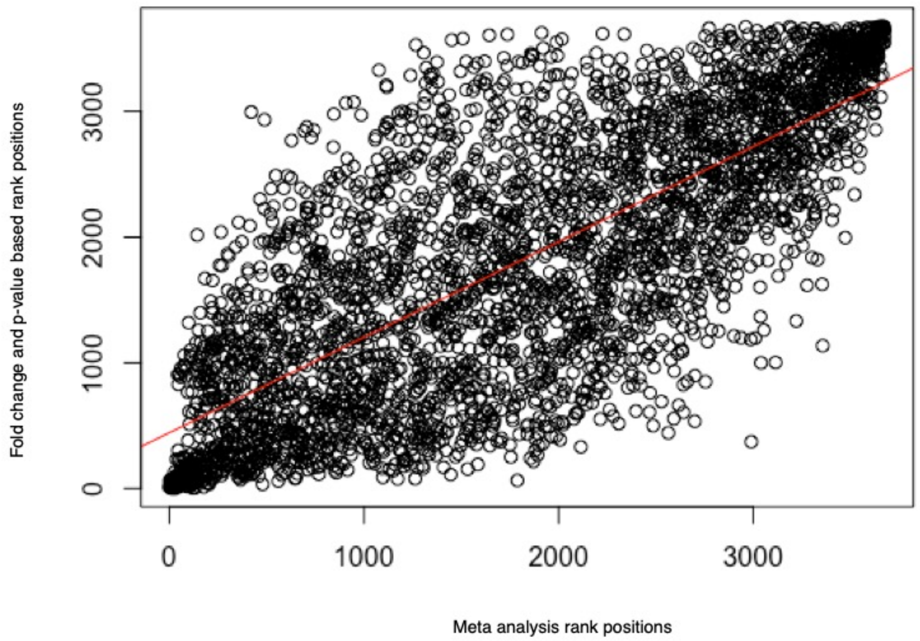

Supplementary Figure 3: Comparison between the positions of the rank obtained by including the fold change information and the rank generated on purely statistical significance. P-value of the gene expression was estimated in the original publications with a a moderatet t-test in the limma package R. The red line represents the fit between the two ranks. The correlation value is 0.7585.

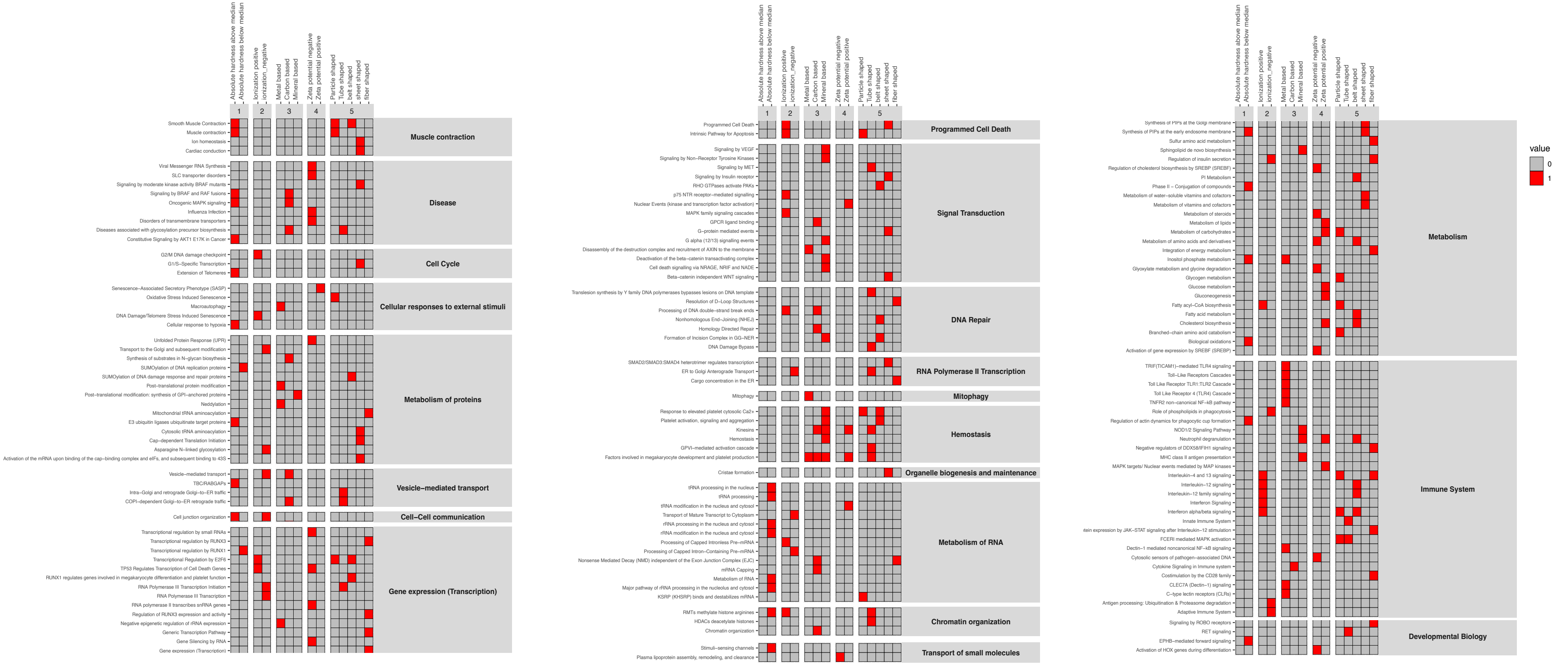

Supplementary Figure 4: Functional annotation of individual molecular signatures from ENMs sharing the same pysichochemical characteristics. Briefly, absolute hardness relates to the stability of the nanoparticle. It represents a measure of reactivity or dissolution, as the harder the material, the more difficult it is to dissolve. We find that nanomaterials with absolute hardness above median have a molecular signature pointing cellular response to stimuli, diseases, muscle contraction, and more on the molecular level, to vesicle mediated transport, probably suggesting they are more bio-persistent and toxic for the biological system. Metal nanoparticles and ion releasing materials have a specific impact on ionic homeostasis which reflects on their mechanism of action and toxicological endpoint. In this molecular signature, we find that the metal-based materials have an impact on elements of the innate immune system, such as the toll-like receptors. Similarly, ion releasing materials affect the immunological functions but mainly by affecting interleukins. In general ionization positive materials also enrich the cell death pathway, probably suggesting that they have a higher toxicity potential. With respect to other basic chemistries, mineral based materials seem to affect the basic homeostasis of the cell, both in calcium signalling and kinesis. In terms of shapes, tubes and belts alter DNA repair mechanisms more specifically. Finally, zeta potential is a measure of the interaction of the material with the environment. Zeta potential negative materials alter pathways associated with viral diseases. On the contrary, zeta potential positive materials enriched the neutrophil degranulation pathway.

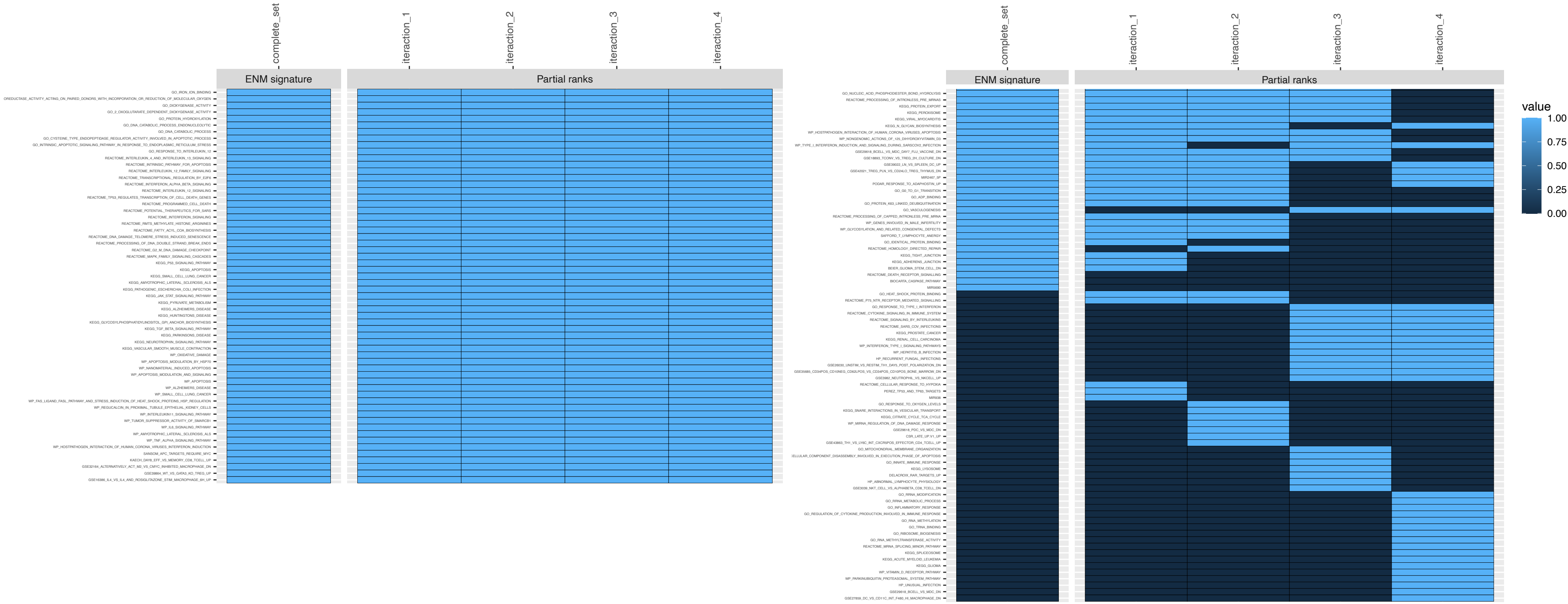

Supplementary Figure 5: Functional annotation of intermediate ENM metanalysis ranks obtained when removing ion releasing materials. Iteraction 1 removes only the ionic datasets. Iteraction 2 removes silver exposures and ionic datasets. Iterations 3 and 4 further remove zinc, copper, cadmium and iron based materials, whether they are pristine or functionalised, respectively. For each rank, a gene set enrichment analysis (GSEA) had been performed on five databases (c.f. Methods), and the top 20 results have been pooled together. When the pathway is enriched, it is scored 1 in the specific column, and represented by light blue. When the pathway is not enriched, it is scored 0 in the specific column and represented in dark blue. When the ion datasets are removed, the vasculogenesis, caspase, death receptor and homology directed repair pathways are no longer enriched in the top positions. When also silver datasets are excluded, tight and adherent junction pathways are not present, as well as the protein dimerization pathway. Changes in protein related pathways (ADP binding, deubiquitination) are similarly emerging when pristine metals are removed from the initial set. Furthermore, the T lymphocytes pathway is no longer in the top positions. Finally, when also functionalised metals are removed, the neuron apoptotic process, necrosis, intronless pre-mRNA, protein export, peroxisome, and viral myocarditis pathways are no longer observed in the top 20 enriched set.

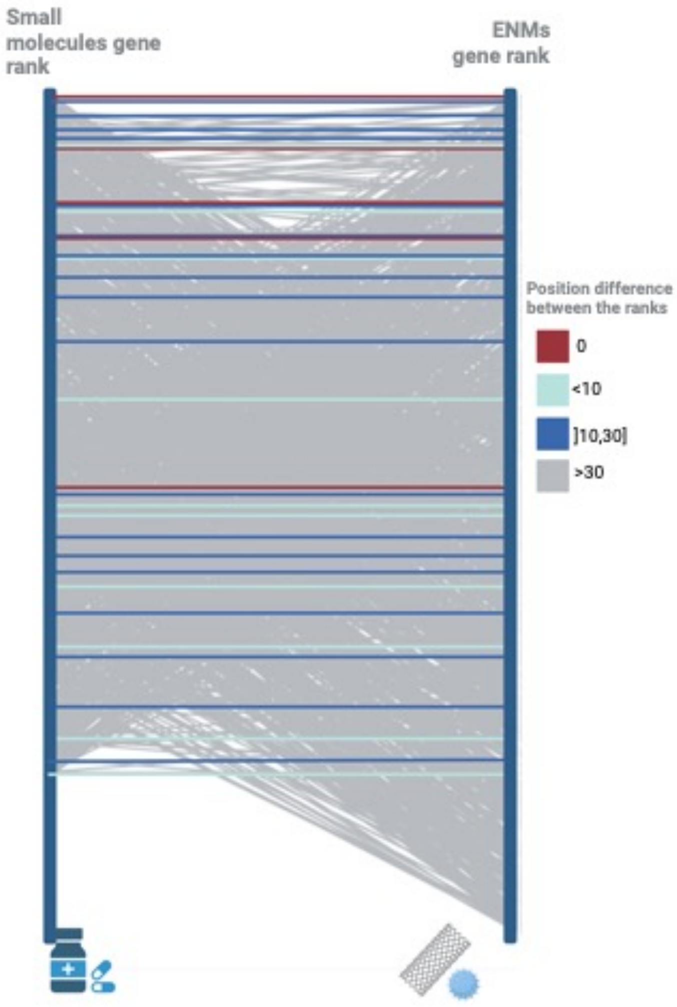

Supplementary Figure 6: Comparison of the meta-analysis ranks obtained from ENMs and drug safety derived data (TG-Gates). Genes sharing the same position in both datasets are marked in red. Genes whose position difference is smaller than 10, between 10 and 30, or bigger than 30, are marked in light green, blue, and grey, respectively.

A

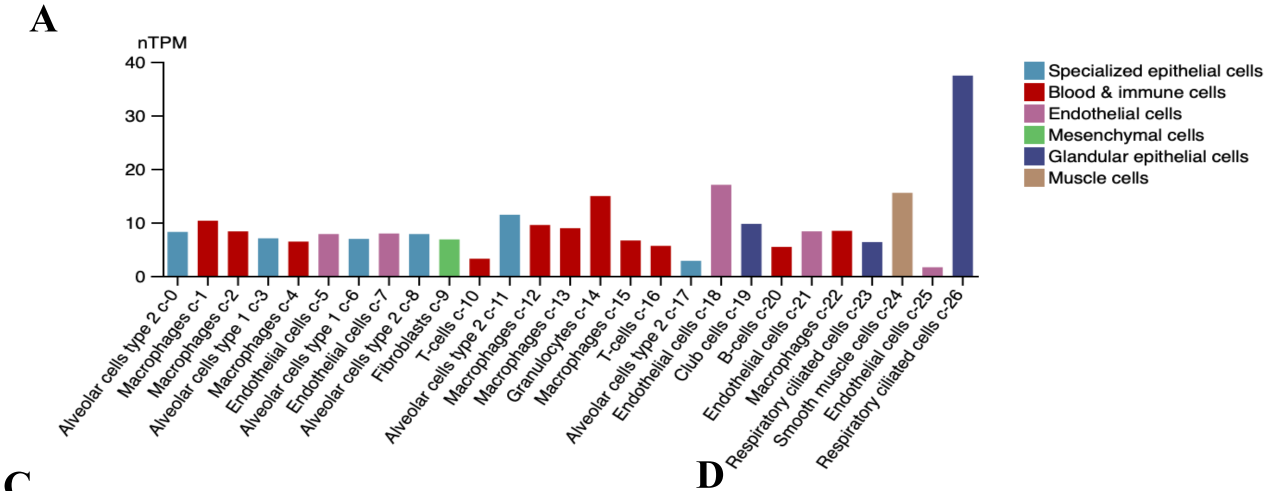

B

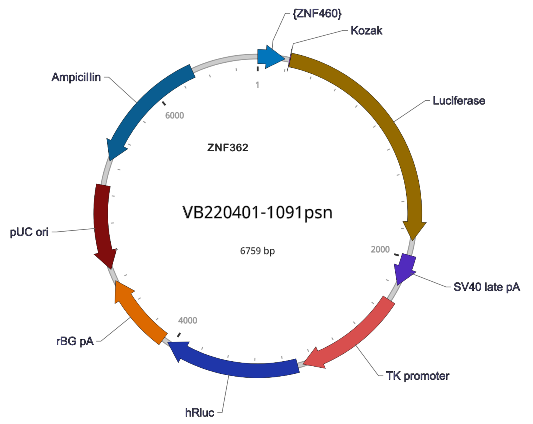

C

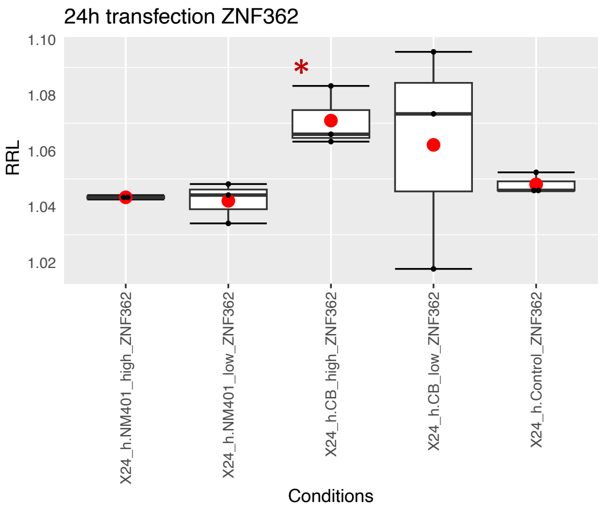

D

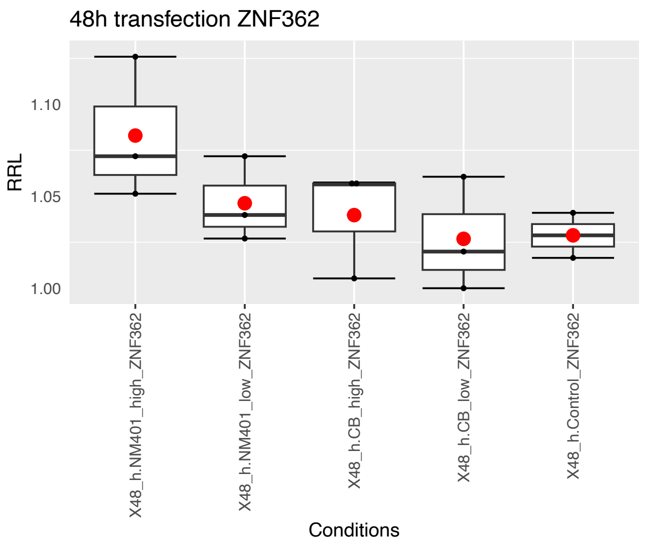

E

Class	mean	median	sd	min	max	whis_low	whis_high
CB_high_ZNF362_24h	1,070939	1,066043	0,010851	1,063398	1,083375	1,064721	1,074709
CB_low_ZNF362_24h	1,062231	1,073346	0,040091	1,017756	1,095592	1,045551	1,084469
Control_ZNF362_24h	1,048046	1,045915	0,003757	1,045838	1,052384	1,045877	1,04915
NM401_high_ZNF362_24h	1,043422	1,043422	0,001088	1,042652	1,044191	1,043037	1,043806
NM401_low_ZNF362_24h	1,042168	1,044239	0,007279	1,034078	1,048187	1,039159	1,046213
CB_high_ZNF362_48h	1,039758	1,056438	0,029806	1,005346	1,057491	1,030892	1,056964
CB_low_ZNF362_48h	1,026849	1,01993	0,030896	1	1,060618	1,009965	1,040274
Control_ZNF362_48h	1,028755	1,028755	0,017319	1,016508	1,041001	1,022631	1,034878
NM401_high_ZNF362_48h	1,083012	1,071773	0,038499	1,051384	1,12588	1,061579	1,098827
NM401_low_ZNF362_48h	1,046205	1,039823	0,02305	1,027018	1,071773	1,033421	1,055798

Supplementary Figure 7: A) Expression of ZNF362 in lung derived cell lines (taken from https://www.proteinatlas.org/) **B**) Vector design as obtained by the provider. **C**) Luciferase signal values recorded after 24 hours from the exposure with two concentrations of NM401 and carbon black, respectively. Beas-2B was transfected with vectors containing ZNF362 promoter prior the exposure. The signal of the control (transfected with the vector) without exposure has been reported as "Control". The signal values have been normalised as described in the methods. Each bar has n=3 replicates (when outliers are not detected). Whiskers of the box plot represent the maximum and minimum values in the data excluding outliers. **D**) The same annotation has been used for Luciferase signal values recorded 48 hours after exposure. The data are presented as mean values (red dots) ± standard error. of n=3 independent biological replicates (when no outliers was detected). Statistical significance was determined using the t-test; The red asterisk indicates when the difference between exposures and control has a p-value equal to 0.05. **E)** Boxplot in C-D have been defined terms of minima, maxima, centre, bounds of box and whiskers and percentile.

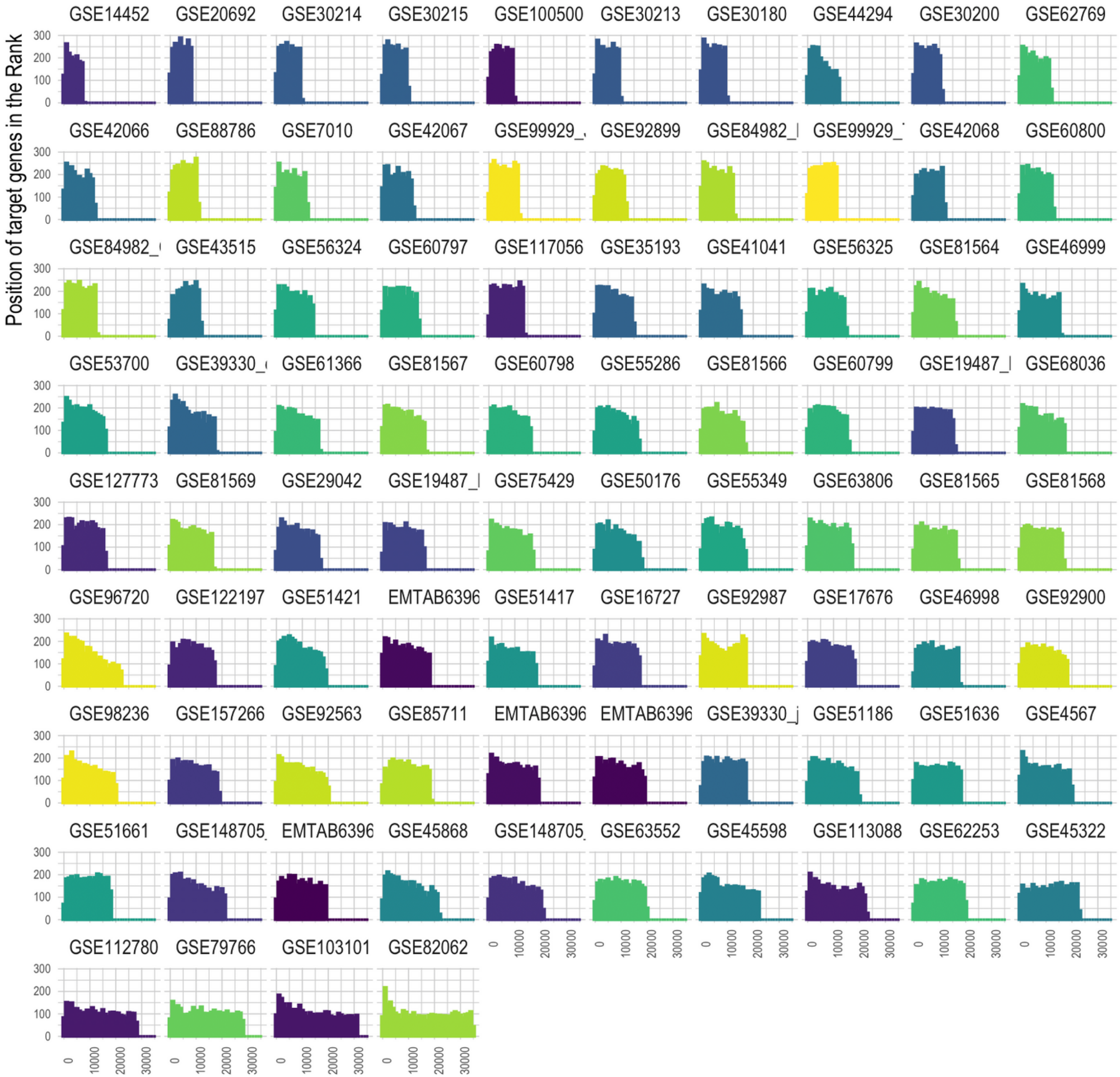

Supplementary Figure 8: Distribution of the transcripts regulated by the identifies transcription factprs in each dataset included and excluded from the initial collection of Saarimäki et al. Transcripts are ranked according to the p-value of the detected alteration. P-value of the gene expression was estimated in the original publications with a a moderatet t-test in the limma package R.

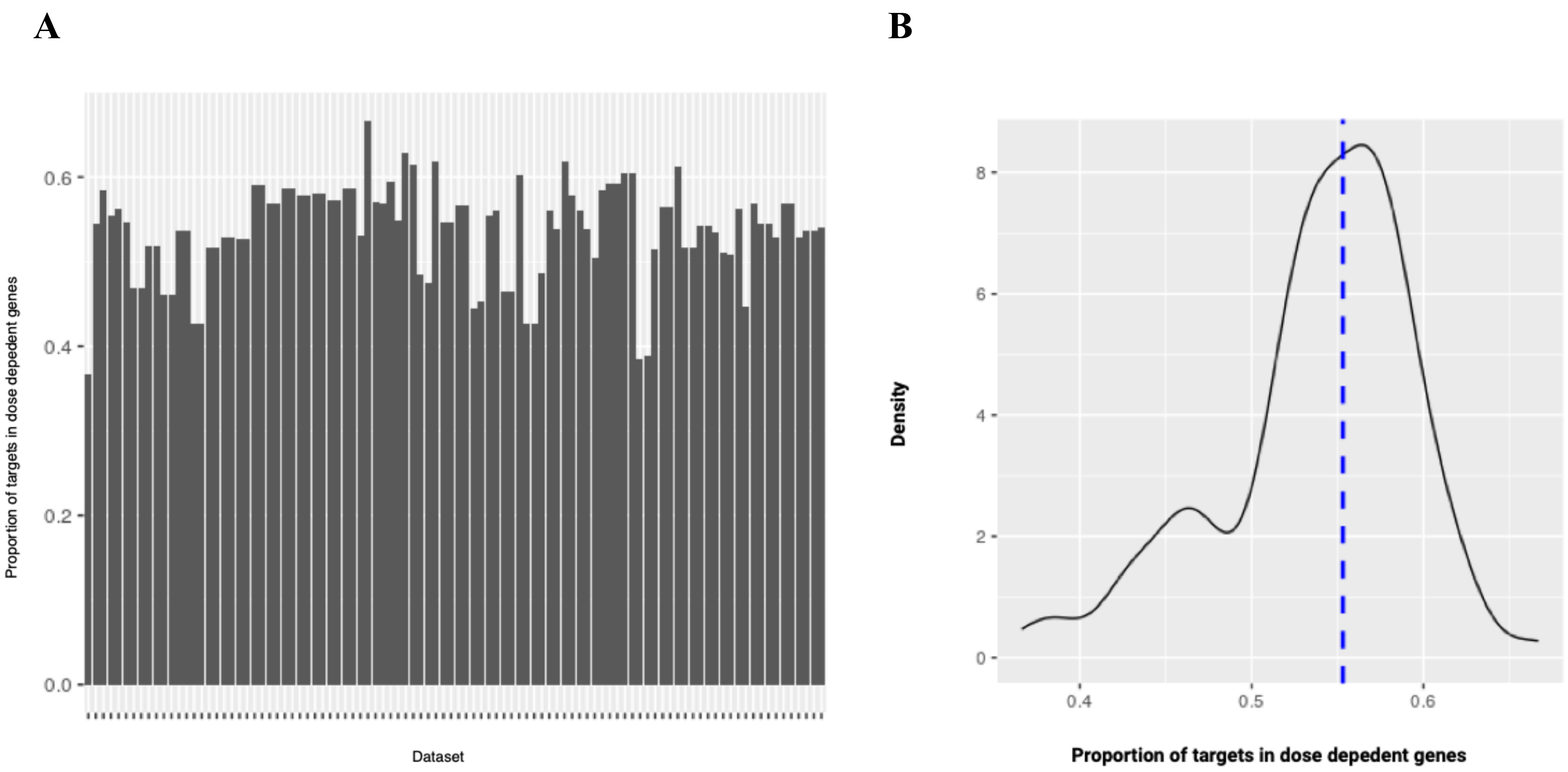

Supplementary Figure 9: A. Proportion of C_2H_2-ZNF regulated genes in the list of dose dependent genes for each dataset **B.** Density plot of the data in A, indicating the mode value of C_2H_2-ZNF coverage in dose dependent genes (blue dashed line).

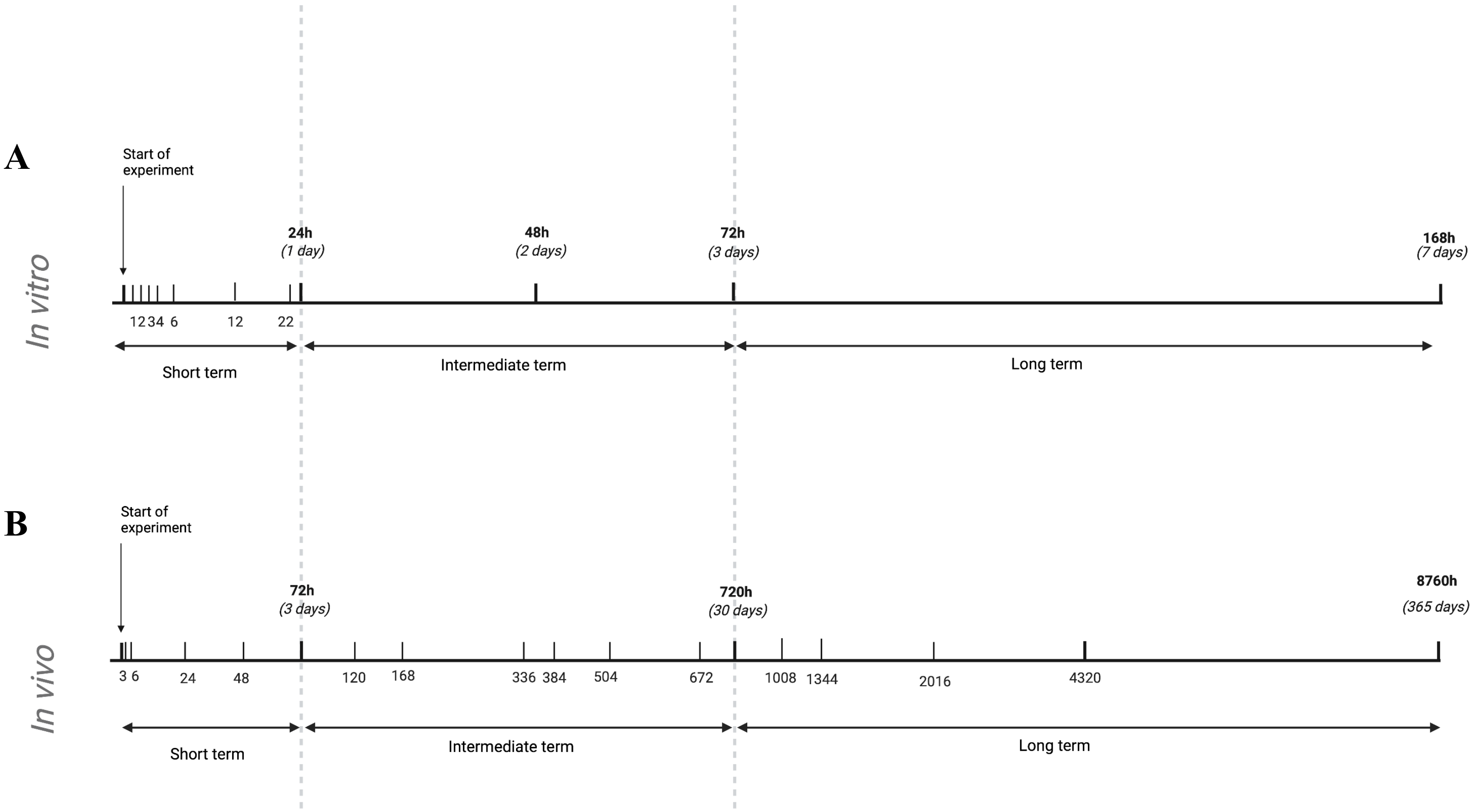

Supplementary Figure 10: Classification of the durations of exposure in *in vitro* **(A)** and *in vivo* **(B)** experimental settings, respectively.
