# Supplementary material for: An ancestral molecular response to nanomaterial particulates
Source: Nat Nanotechnol. 2023 May 8;18(8):957–66. doi: 10.1038/s41565-023-01393-4 (PMC10427433; doi:10.1038/s41565-023-01393-4)
Supplement: Supplementary file 2 — Reporting Summary [file 41565_2023_1393_MOESM2_ESM.pdf]

nature portfolio

Corresponding author(s): Dario Greco

Last updated by author(s): Mar 17, 2023

Reporting Summary

Nature Portfolio wishes to improve the reproducibility of the work that we publish. This form provides structure for consistency and transparency in reporting. For further information on Nature Portfolio policies, see our Editorial Policies and the Editorial Policy Checklist.

Statistics

For all statistical analyses, confirm that the following items are present in the figure legend, table legend, main text, or Methods section.

n/a	Confirmed	
☐	☒	The exact sample size (*n*) for each experimental group/condition, given as a discrete number and unit of measurement
☐	☒	A statement on whether measurements were taken from distinct samples or whether the same sample was measured repeatedly
☐	☒	The statistical test(s) used AND whether they are one- or two-sided<br>*Only common tests should be described solely by name; describe more complex techniques in the Methods section.*
☐	☒	A description of all covariates tested
☐	☒	A description of any assumptions or corrections, such as tests of normality and adjustment for multiple comparisons
☐	☒	A full description of the statistical parameters including central tendency (e.g. means) or other basic estimates (e.g. regression coefficient) AND variation (e.g. standard deviation) or associated estimates of uncertainty (e.g. confidence intervals)
☐	☒	For null hypothesis testing, the test statistic (e.g. *F*, *t*, *r*) with confidence intervals, effect sizes, degrees of freedom and *P* value noted<br>*Give P values as exact values whenever suitable.*
☒	☐	For Bayesian analysis, information on the choice of priors and Markov chain Monte Carlo settings
☒	☐	For hierarchical and complex designs, identification of the appropriate level for tests and full reporting of outcomes
☐	☒	Estimates of effect sizes (e.g. Cohen's *d*, Pearson's *r*), indicating how they were calculated

Our web collection on statistics for biologists contains articles on many of the points above.

Software and code

Policy information about availability of computer code

Data collection	No software was used to download omics data. The original publications manuscripts were downloaded with Biopython API (www.biopython.org).
Data analysis	Preprocessing of transcriptomic data has been performed using eUTOPIA (https://github.com/Greco-Lab/eUTOPIA) version commit December 2021. Dose dependent analysis was performed using the BMDx tool (https://github.com/Greco-Lab/BMDx) version commit February 2022. Functional enrichment was performed with the FunMappOne tool (https://github.com/Greco-Lab/FunMappOne) version commit December 2021. Computation of molecular descriptors has been performed with the semi-empirical code MOPAC version PM6 (http://OpenMOPAC.net) , the SIESTA code (as described by Soler et al.) and the GFN-xTB method (reported in Bannwarth et al.). Promoter analysis was performed with the MEME suite version 5.5.1 (https://meme-suite.org/meme/) and annotation to transcription factor binding sites was performed with FactorBook (https://www.factorbook.org, version 1). All the custom analysis and scripts have been generated in the R environment version 4.0.5. The following R packages have been used: esc, version 0.5.1, metap version 1.8, RankProd version 3.24.0, TopKList version 1.0.8, Fgsea version 1.22.0, stats version 4.2.0, DescTools version 0.99.43, limma version 3.52.4, Biomart version 2.52.0, Affy version 1.60.0 . The code has been deposited in the online Zenodo repository (https://doi.org/10.5281/zenodo.7674574) and on Github under https://github.com/fhaive/metanalysis_toxicogenomic_data.

For manuscripts utilizing custom algorithms or software that are central to the research but not yet described in published literature, software must be made available to editors and reviewers. We strongly encourage code deposition in a community repository (e.g. GitHub). See the Nature Portfolio guidelines for submitting code & software for further information.

Data

Policy information about availability of data

All manuscripts must include a data availability statement. This statement should provide the following information, where applicable:

- Accession codes, unique identifiers, or web links for publicly available datasets
- A description of any restrictions on data availability
- For clinical datasets or third party data, please ensure that the statement adheres to our policy

The preprocessed version of the transcriptomic datasets included in the discovery datasets, i.e., ENM exposures of human and mouse samples, have been previously deposited at https://zenodo.org/record/3949890#.YlPUri0RqH0. The original datasets can be accessed at Array Express (https://www.ebi.ac.uk/biostudies/arrayexpress) with the entry code EMTAB6396, and at GEO (https://www.ncbi.nlm.nih.gov/) under accession number GSE103101, GSE112780, GSE113088, GSE117056, GSE122197,GSE127773,GSE148705, GSE157266, GSE16727, GSE17676, GSE19487, GSE20692, GSE29042, GSE35193, GSE39330, GSE41041, GSE42066, GSE42067, GSE42068, GSE43515, GSE45322, GSE45598, GSE4567, GSE46998, GSE46999, GSE50176, GSE51186, GSE51417, GSE51421, GSE51636, GSE53700, GSE55286, GSE55349, GSE56324, GSE56325, GSE60797, GSE60798, GSE60799, GSE60800, GSE61366, GSE62253, GSE62769, GSE63552, GSE63806, GSE68036, GSE75429, GSE79766, GSE81564, GSE81565, GSE81566, GSE81567, GSE81568, GSE81569, GSE82062, GSE84982, GSE85711, GSE88786, GSE92563, GSE92900, GSE92987, GSE96720, GSE98236, GSE99929.
Transcriptomic datasets used for the eco-toxicological analysis are freely available at GEO under accession numbers GSE80461, GSE32521, GSE70509, GSE73427, GSE77148, GSE41333, GSE47662. Drug exposed datasets have been downloaded from https://dbarchive.biosciencedbc.jp/en/open-tggates/download.html in November 2020. Functional data were downloaded from https://www.gsea-msigdb.org/gsea/msigdb/ version 7.2.
All the other relevant data and supporting the findings of this study have been deposited in the online Zenodo repository (https://doi.org/10.5281/zenodo.7674574).

Human research participants

Policy information about studies involving human research participants and Sex and Gender in Research.

Reporting on sex and gender	does not apply
Population characteristics	does not apply
Recruitment	does not apply
Ethics oversight	does not apply

Note that full information on the approval of the study protocol must also be provided in the manuscript.

Field-specific reporting

Please select the one below that is the best fit for your research. If you are not sure, read the appropriate sections before making your selection.

☒ Life sciences ☐ Behavioural & social sciences ☐ Ecological, evolutionary & environmental sciences

For a reference copy of the document with all sections, see nature.com/documents/nr-reporting-summary-flat.pdf

Life sciences study design

All studies must disclose on these points even when the disclosure is negative.

Sample size	This study included 68 datasets of ENMs exposure to human and mouse previously manually curated and preprocessed according to standardized protocols (https://zenodo.org/record/3949890#.YlPUri0RqH0 , Saarimäki et al. DOI: 10.1038/s41597-021-00808-y). From the datasets we studied 584 experimental instances (ENM-dose-time point specific) and covered the expression of 3,676 genes. As for the drug exposures, expression of the same genes has been explored in 150 individual exposures which were freely available at the Open TGgates datates (https://dbarchive.biosciencedbc.jp/en/open-tggates/download.html). This study used all the ENMs exposure datasets that were available at the moment of the analysis that would be considered FAIR. Therefore this study relies on the largest curated collection of ENMs transcriptomic data currently available at the moment of the study. Indeed, all the datasets included have been selected as satisfying number of replicates (n=3), presence of untreated control samples (negative controls), and rigorous experimental design. Similarly, as for eco-toxicological datasets retrival, upon literature reviews, all datasets of ENMs exposures in non mammal species which would contain differentially expressed and annotated genes were selected for the study. Therefore no statistical methods were used to calculate the samples size because every datasets satisfying the quality requirements was taken into account.
Data exclusions	Datasets have been excluded in case the microarray platform used in the experiment was not commercially available (custom designed), or marginally represented.
Replication	Original omic data was performed in at least triplicates successfully. The raw files of the original files underwent strict quality control and only the samples passing the quality standard were included (Saarimäki et al. DOI: 10.1038/s41597-021-00808-y). Statistical evaluation of our results was internally performed through the application of the meta-analitical approaches, which represents a quantitative synthesis of the

	individual experimental instances. Furthermore the results pointing to the zinc fingers models were successfully replicated in multiple datasets and species (GSE80461, GSE32521, GSE70509, GSE73427, GSE77148, GSE41333, GSE47662 and https://dbarchive.biosciencedbc.jp/en/open-tggates/download.html), supporting the reproducibility of this study.
Randomization	Randomization protocols for omics data were described in the original publications reporting each dataset (EMTAB6396,GSE103101, GSE112780, GSE113088, GSE117056, GSE122197,GSE127773,GSE148705, GSE157266, GSE16727, GSE17676, GSE19487, GSE20692, GSE29042, GSE35193, GSE39330, GSE41041, GSE42066, GSE42067, GSE42068, GSE43515, GSE45322, GSE45598, GSE4567, GSE46998, GSE46999, GSE50176, GSE51186, GSE51417, GSE51421, GSE51636, GSE53700, GSE55286, GSE55349, GSE56324, GSE56325, GSE60797, GSE60798, GSE60799, GSE60800, GSE61366, GSE62253, GSE62769, GSE63552, GSE63806, GSE68036, GSE75429, GSE79766, GSE81564, GSE81565, GSE81566, GSE81567, GSE81568, GSE81569, GSE82062, GSE84982, GSE85711, GSE88786, GSE92563, GSE92900, GSE92987, GSE96720, GSE98236, GSE99929). Samples in this study have not be allocated in experimental groups. As the aim of the study was to highlight commonalities between ENMs, this study does not perform any statistical analysis between groups but considers all exposures as ENMs based. A thorough characterization of the experimental samples has been provided and is based on the experimental information reported in the original studies and the physichochemical properties of the ENMs as reported in the original publications.
Blinding	Blinding techniques were not needed for this study. Blinding techniques are needed to minimize allocation bias. As explained, samples in this study have not been allocated in experimental groups. All samples have been considered as belonging to the ENMs groups.

Reporting for specific materials, systems and methods

We require information from authors about some types of materials, experimental systems and methods used in many studies. Here, indicate whether each material, system or method listed is relevant to your study. If you are not sure if a list item applies to your research, read the appropriate section before selecting a response.

Materials & experimental systems

n/a	Involved in the study
☒	☐ Antibodies
☐	☒ Eukaryotic cell lines
☒	☐ Palaeontology and archaeology
☒	☐ Animals and other organisms
☒	☐ Clinical data
☒	☐ Dual use research of concern

Methods

n/a	Involved in the study
☒	☐ ChIP-seq
☒	☐ Flow cytometry
☒	☐ MRI-based neuroimaging

Eukaryotic cell lines

Policy information about cell lines and Sex and Gender in Research

Cell line source(s)	The human acute monocytic leukemia cell line THP-1 was purchased from the American Type Culture Collection (ATCC).
Authentication	The cells were used for up to 30 passages and were tested regularly using MycoAlert® mycoplasma detection kit (Lonza).<br><br>THP-1 cells were not authenticated by us but ATCC has performed cell line authentication by using STR analysis/profiling.
Mycoplasma contamination	All cell lines were tested negative for mycoplasma
Commonly misidentified lines<br>(See ICLAC register)	None
